# Climbing fibers provide essential instructive signals for associative learning

**DOI:** 10.1038/s41593-024-01594-7

**Published:** 2024-04-02

**Authors:** N. Tatiana Silva, Jorge Ramírez-Buriticá, Dominique L. Pritchett, Megan R. Carey

**Affiliations:** 1 Neuroscience Program, Champalimaud Center for the Unknown, Lisbon, Portugal; 2 Biology Department, Howard University, Washington, DC USA

**Keywords:** Neural circuits, Learning and memory

## Abstract

Supervised learning depends on instructive signals that shape the output of neural circuits to support learned changes in behavior. Climbing fiber (CF) inputs to the cerebellar cortex represent one of the strongest candidates in the vertebrate brain for conveying neural instructive signals. However, recent studies have shown that Purkinje cell stimulation can also drive cerebellar learning and the relative importance of these two neuron types in providing instructive signals for cerebellum-dependent behaviors remains unresolved. In the present study we used cell-type-specific perturbations of various cerebellar circuit elements to systematically evaluate their contributions to delay eyeblink conditioning in mice. Our findings reveal that, although optogenetic stimulation of either CFs or Purkinje cells can drive learning under some conditions, even subtle reductions in CF signaling completely block learning to natural stimuli. We conclude that CFs and corresponding Purkinje cell complex spike events provide essential instructive signals for associative cerebellar learning.

## Abstract (web summary)

Silva et al. definitively establish climbing fiber-driven complex spike events as essential instructive signals for associative cerebellar learning while also revealing unexpected features of optogenetic manipulation.

## Main

Instructive signals are a core component of supervised learning systems. In the brain they are thought to be conveyed by specific classes of neurons that trigger modification of neural pathways that control behavior. CF projections from the inferior olive to the cerebellar cortex have long been hypothesized to carry neural instructive error signals for various forms of learning, including associative eyeblink conditioning and several forms of motor adaptation^1–9^.

**Fig. 1:**
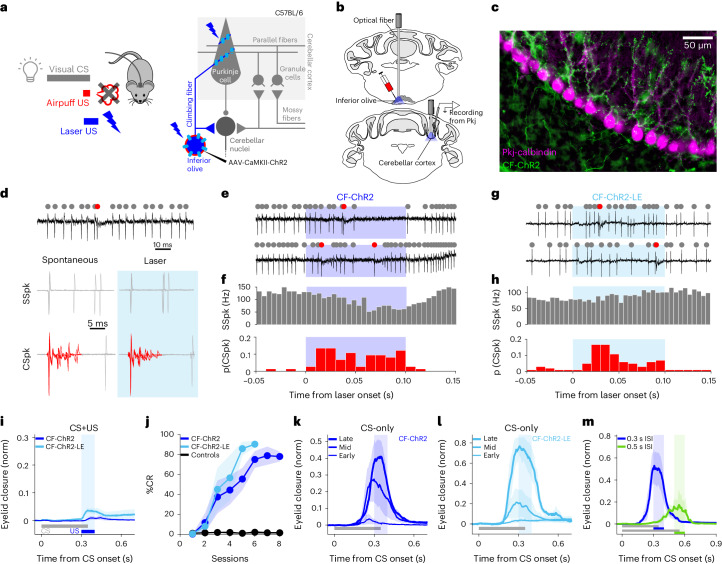
Optogenetic CF stimulation instructs eyeblink conditioning. **a**, Left: experimental scheme. The traditional airpuff US was replaced by laser stimulation and paired with a visual CS. Right: cerebellar circuit and experimental strategy. **b**, Optical fibers implanted in either the left IO or right eyelid region of the cerebellar cortex, where Purkinje cells were recorded. **c**, Example sagittal section of cerebellar cortex (similar expression was observed in 15 mice). ChR2 (green) expression in CF inputs to Purkinje cells (magenta) (Extended Data Fig. 1). **d**, SSpk and CSpk Purkinje cell waveforms during spontaneous and laser epochs. **e**, Electrophysiological traces from a Purkinje cell showing SSpks (gray dots) and CSpks (red dots) during CF-ChR2 laser stimulation in IO (CF-ChR2-IO, blue). **f**, Population histogram of SSpk firing rate (gray) and CSpk probability (p(CSpk)) (red) (*n* = 74 trials, *N* = 4 units from 2 mice). CSpks: spontaneous versus laser, ^*^P = 0.02, paired Student’s *t*-test; SSpks: spontantaneous versus laser, *P* = 0.22 nonsignificant (NS), paired Student’s *t*-test. **g**,**h**, As for **e** and **f**, respectively, but for CF-ChR2-LE animals (CF-ChR2-LE-IO; *n* = 102 trials, *N* = 8 cells from 2 mice). CSpks: spontaneous versus laser, ^*^P = 0.01, paired Student’s *t*-test; SSpks: spontaneous versus laser, *P* = 0.11 NS, paired Student’s *t*-test. **i**, Average eyelid closure traces ± s.e.m. (shadows) from CS + US trials of the first training session showing no reflexive eyeblink to CF-ChR2-IO stimulation (*N* = 7 mice, blue) and very small eye twitch in CF-ChR2-LE-IO animals (*N* = 4 mice, light blue). norm, normalized. **j**, The %CR across daily training sessions ± s.e.m. (shadows) for CF-ChR2-IO (*N* = 7 mice, blue) or CF-ChR2-LE-IO (*N* = 4 mice, light blue) laser US training. Controls: wild-type mice (no ChR2 expression) with fiber in IO and laser US (*N* = 2 mice, black). The %CR at the last learning session (all two-sample Student’s *t*-tests): CF-ChR2-IO versus controls, ^***^P = 1.7726 × 10^−4^ (7 versus 2 mice); CF-ChR2-LE-IO versus controls, ^***^P = 4.0836 × 10^−5^ (4 versus 2 mice); CF-ChR2-IO versus CF-ChR2-LE-IO, *P* = 0.115 NS (7 versus 4 mice). **k**, Average eyelid closures ± s.e.m. (shadows) from CS-only trials of sessions 2, 4 and 8 for CF-ChR2-IO experiments shown in **j**. The shaded rectangle indicates time US would have appeared. **l**, Same as **k** but for sessions 2, 4 and 6 of CF-ChR2-LE-IO. **m**, Average eyelid closures ± s.e.m. (shadows) from CS-only trials after training to a 300-ms (blue, *N* = 4 mice) and 500-ms (green, *N* = 4 mice) CS + US ISI. Peak time: ^*^P = 0.01, paired Student’s *t*-test.

According to the CF hypothesis, CF activity drives associative plasticity at parallel fiber inputs to cerebellar Purkinje cells, which forms the neural substrate for learning. There are several lines of evidence in support of this hypothesis. In contrast to typical ‘simple spikes’ (SSpks), which are driven by excitatory parallel fiber inputs, CFs evoke powerful ‘complex spikes’ (CSpks) in cerebellar Purkinje cells (Fig. 1a–d). Complex spikes have a unique electrophysiological signature revealing multiple ‘spikelets’ (Fig. 1d). They are associated with elevations in dendritic calcium and drive heterosynaptic plasticity at parallel fiber-to-Purkinje cell synapses^10–14^. CSpk activity is associated with sensorimotor errors for a range of behavioral tasks, with the probability of a CSpk often changing in predictable ways with the development of learning^15–22^ and its extinction^23–25^. Moreover, electrical stimulation of CF pathways is sufficient to substitute for an airpuff unconditioned stimulus (US) to drive eyeblink conditioning in rabbits^26,27^ and recent experiments have shown that optogenetic CF stimulation can trigger adaptation of the vestibulo-ocular reflex^28,29^ (VOR), whereas inhibition of CFs can drive extinction of eyeblink conditioning^24,25^.

However, substantial confusion and controversy remain, particularly with regard to the necessity of CF instructive signals and CSpk-driven plasticity for learning^8,30–35^. For instance, there is substantial experimental support for an alternative model that posits that Purkinje cell SSpk modulation, rather than CF-driven CSpks, could provide relevant instructive signals for learning^4,36^. This hypothesis stems from the observation that sensorimotor errors that drive CF activity and subsequent Purkinje cell CSpks are often tightly linked to rapid, reflexive, corrective movements. Crucially, Purkinje cell SSpk output often correlates with these corrective movements^34,37,38^, raising the possibility that they could provide their own instructive signals for plasticity—either in addition to, or independently of, CSpk activity^30,36,39–44^. In particular, Purkinje cell SSpk modulation could instruct plasticity in the downstream cerebellar nuclei^5,36^, an idea that also has support from in vitro experiments of synaptic plasticity^45,46^.

Seemingly consistent with a possible instructive role for Purkinje cell SSpk modulation, recent work has demonstrated that pairing optogenetic stimulation of Purkinje cells, which effectively modulates SSpk activity, with ongoing movements can drive motor adaptation in multiple systems^28,47,48^. However, it is not clear whether this optogenetically evoked learning results from modulation of SSpk output and/or from the generation of CSpk-like dendritic calcium signals in Purkinje cells that instruct plasticity in the cerebellar cortex^48^.

Just as it has not been clear whether Purkinje cell SSpks could provide alternative instructive signals, it has also not been clear whether CF signaling is absolutely required for cerebellar learning. Although Purkinje cell CSpk activity often correlates with sensorimotor errors that drive behavioral learning, the extremely low rates of CSpk activity and high proportion of ‘spontaneous’ CSpks that appear not to correspond with identifiable task parameters complicate a definitive interpretation of CSpks as instructive signals^34^. Moreover, much of the evidence to date that has been interpreted as supporting a causal role for CF instructive signals for cerebellar learning has come from lesion studies^49^, pharmacological inactivations^24,31^ and electrical perturbations^50,51^ of the inferior olive (IO). These manipulations lack both cell-type and temporal specificity and are likely to have substantial, additional, unintended effects on the olivocerebellar circuit^52^ that are extremely difficult to control for. Until now, there has not been a precise way to selectively perturb evoked CF activity while leaving olivocerebellar function otherwise intact.

In the present study, we used cell-type-specific perturbations of CFs, Purkinje cells and other circuit elements to test their sufficiency and necessity as instructive signals for associative cerebellar learning. We combined behavioral, optogenetic and electrophysiological approaches to dissociate CF inputs and CSpk activity from reflexive movements and SSpk modulation. We find that optogenetically evoked CSpks can substitute for an airpuff US to induce learning, even in the absence of an evoked blink, whereas temporally precise optogenetic silencing of CFs completely blocks learning. Direct optogenetic stimulation of Purkinje cells can also drive learning; however, this effect was dissociable from both SSpk modulation and the corresponding evoked blink. Finally, simple ChR2 expression in CFs is associated with a subtle decrease in Purkinje cell CSpk probability that abolishes learning to a sensory US. Together, our results support a necessary and sufficient role for CFs and corresponding Purkinje cell CSpk events as instructive signals for associative cerebellar learning.

## Results

We investigated neural instructive signals for delay eyeblink conditioning in head-fixed mice walking on a motorized treadmill^53,54^. In classic eyeblink conditioning experiments (Fig. 1a), a neutral conditioned stimulus (CS; here a white light light-emitting diode (LED)) is paired with a US (usually a puff of air directed at the eye) that reliably elicits an eyeblink unconditioned response (UR) and serves as an instructive signal for learning. CFs from the dorsal accessory part of the IO respond to the airpuff US and project to the contralateral cerebellum, where they drive CSpks in Purkinje cells in the cerebellar cortex (Fig. 1a,b). Information about the CS is conveyed to the cerebellum by mossy fibers that synapse on to granule cells, the axons of which form parallel fiber inputs that modulate SSpks in Purkinje cells. Pauses in SSpk activity in the eyelid region of the cerebellar cortex are associated with eyelid closures^55–58^. In the present study, we use genetic circuit dissection to distinguish between competing models in which CSpk and/or SSpk modulation provides instructive signals for eyeblink conditioning. We first asked whether direct optogenetic stimulation of CFs could substitute for a sensory (airpuff) US to drive behavioral learning (Fig. 1).

### Optogenetic CF stimulation is sufficient to drive learning

**Extended Data Fig. 1:**
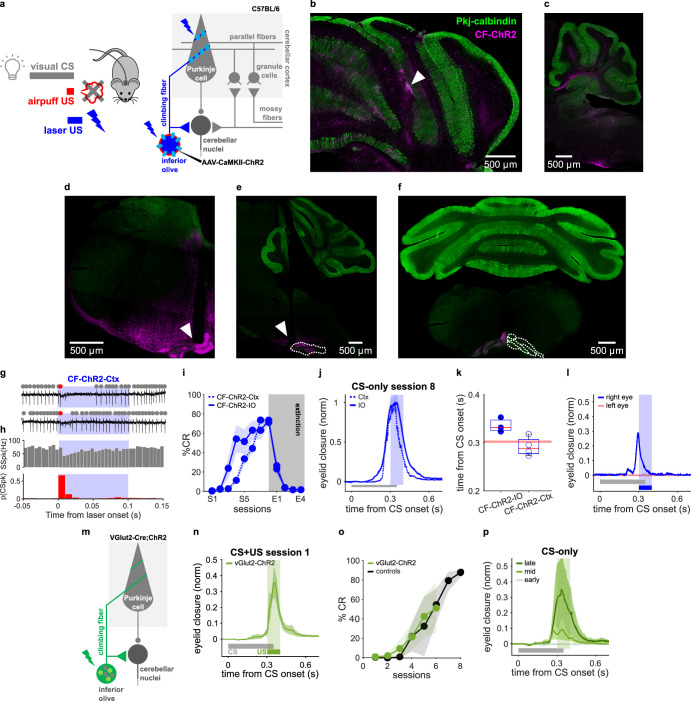
Multiple genetic and anatomical strategies for targeting IO neurons. **a**, Experimental scheme. **b-f**, Histological examples representative of 11 CF-ChR2 mice with IO fiber, 4 with Ctx fiber. **b**, Coronal section showing fiber placement (white arrow), in eyelid area of right cerebellar cortex. **c**, Sagittal section showing ChR2 expression in climbing fiber projections to the cerebellar cortex. **d**, Coronal, and **e**, sagittal sections showing CF-ChR2 expression and optical fiber placement (white arrow) in left IO. **f**, Coronal section showing ChR2 expression in left IO. **g**, Electrophysiological traces from a Purkinje cell with identified SSpks (grey dots) and CSpks (red) during CF-ChR2 laser stimulation in cerebellar cortex (CF-ChR2-Ctx, blue). **h**, Population histogram of SSpks (grey) and CSpks (red) (n = 211 trials, N = 15 units from 5 mice). CSpks: spont. vs laser, P = 1.82e-06***, paired t-test; SSpks: spont. vs laser, **P = 0.002, paired t-test. **i**, %CR + -SEM over training (S1-S8) and extinction sessions (E1-E4) of animals with CF-ChR2 stimulation in IO (CF-ChR2-IO, solid line, N = 3 mice) or cerebellar cortex (CF-ChR2-Ctx, dotted line, N = 4 mice) as US. %CR in S8: CF-ChR2-IO vs CF-ChR2-Ctx, P = 0.71n.s., two-sample t-Test (3 vs 4 mice). **j**, Normalized CRs ( ± SEM, shadows) (for experiments in *i*) illustrate broader CR timing for CF-ChR2-IO stimulation, which had sustained CSpk increase (Fig. 1f,h vs Extended Data Fig. 1h). **k**, Eyelid closure timing was subtly later for CF-ChR2-IO. Peak time: CF-ChR2-IO vs CF-ChR2-Ctx, P = 0.37n.s, two-sample t-test (3 vs 4 mice). Each dot is one mouse; box plots indicate median (center bar), 25th-75th percentiles (bottom and top borders), whiskers extend to data extrema. **l**, Conditioning was unilateral (right eye blue, left eye red; N = 2 mice). **m**, Experimental scheme for experiments stimulating glutamatergic IO neurons as US (vGlut2-ChR2-IO). **n**, Average eyelid closures ±SEM (shadows) on CS + US trials in the first training session showing blink evoked by vGlut2-ChR2-IO stimulation (N = 3 mice). **o**, %CR ± SEM for learning to vGlut2-ChR2-IO stimulation (green, N = 3 mice) and controls (expressing ChR2 but without laser stimulation, learning to airpuff-US). %CR at last session: vGlut2-ChR2-IO vs controls, P = 0.94n.s., two-sample t-test (3 vs 3 mice). **p**, Average eyelid closure traces ±SEM (shadows) from CS-only trials of sessions 2,4,6 for vGlut2-ChR2-IO-US (N = 3 mice).

To specifically target CFs, we injected a virus that allows for expression of ChR2 under control of the CaMKIIα promoter^28^ (AAV-CaMKII-ChR2, here termed CF-ChR2) into the dorsal accessory IO of wild-type mice (Fig. 1a,b and Methods). With this strategy, we observed selective labeling of neurons in the IO and CFs in the cerebellar cortex (Fig. 1c and Extended Data Fig. 1a–f). An optical fiber was placed either in the dorsal accessory IO^25,26,28^ (CF-ChR2-IO), targeting cell bodies of eyeblink-related CFs, or in the eyelid region of the cerebellar cortex^56–59^, targeting CF terminals (CF-ChR2-Ctx; Fig. 1b, Extended Data Fig. 1 and Methods). Laser stimulation at both sites evoked robust postsynaptic CSpk responses in cerebellar Purkinje cells, with waveforms matching those of spontaneous CSpks (Fig. 1d–f and Extended Data Fig. 1g,h). Similar electrophysiological responses were observed in mice expressing ChR2 at standard levels or with a slightly reduced viral titer (CF-ChR2-LE; fivefold lower titer; Fig. 1g,h).

To test the sufficiency of CF activity for the acquisition of learned eyelid responses, we paired a neutral visual CS with optogenetic CF stimulation in the absence of any sensory US (CF-ChR2-US; Fig. 1a). Laser stimulation alone did not elicit robust eyelid closures (Fig. 1i). Despite the absence of an eyeblink UR to the optogenetic US, conditioned eyelid closure responses (CRs) gradually emerged in response to the visual CS after repeated CS + US pairing (Fig. 1j,k). Similar learning was observed with both expression levels (Fig. 1j–l) and for fiber placements in either the IO (CF-ChR2-IO; Fig. 1d–l) or the cerebellar cortex (CF-ChR2-Ctx; Extended Data Fig. 1g–k) (although note the subtly different CR and CSpk timings in the two cases). Moreover, learning was also observed in separate experiments in which we targeted ChR2 expression to glutamatergic IO neurons with a transgenic, rather than a viral, strategy, by crossing vGlut2-Cre mice with ChR2-floxed mice^60^ (*vglut2-Cre;ChR2*; Methods) and placing the fiber in the IO (vGlut2-ChR2-IO; Extended Data Fig. 1m–p).

In general, the properties of learning to an optogenetic CF US matched those of normal sensory CS + US conditioning in wild-type mice^53,54^. Learning to an optogenetic US was unilateral (specific to the eye contralateral to the IO and ipsilateral to the corresponding cerebellar cortex; Extended Data Fig. 1l) and emerged over several days, with both frequency and amplitude of learned eyelid closures increasing gradually across sessions (Fig. 1j–l and Extended Data Fig. 1i,o,p); the percentage of trials with CRs (%CR) at the last learning session: CF-ChR2-IO versus airpuff US controls in Extended Data Fig. 1o, *P* = 0.29 NS, Student’s *t*-test; CF-ChR2-LE-IO versus airpuff US controls in Extended Data Fig. 1o, *P* = 0.54 NS, Student’s *t*-test. Learning also extinguished appropriately on cessation of CS + US pairing, when CSs were presented alone (Extended Data Fig. 1i).

A central feature of eyeblink conditioning is the appropriate timing of the CR, so that its peak generally coincides with the expected time of the arrival of the US^61,62^. This appropriate timing was also observed for learning to an optogenetic US (Fig. 1k,l and Extended Data Fig. 1j,k,p). Moreover, when the interval between CS and CF-ChR2 US onset was shifted from 300 ms to 500 ms, mice adapted the timing of their learned responses^54,62^ (Fig. 1m).

These results indicate that optogenetic CF activation is sufficient to substitute for an airpuff US to instruct delayed eyeblink conditioning.

### Optogenetic Purkinje cell stimulation can drive learning

**Fig. 2:**
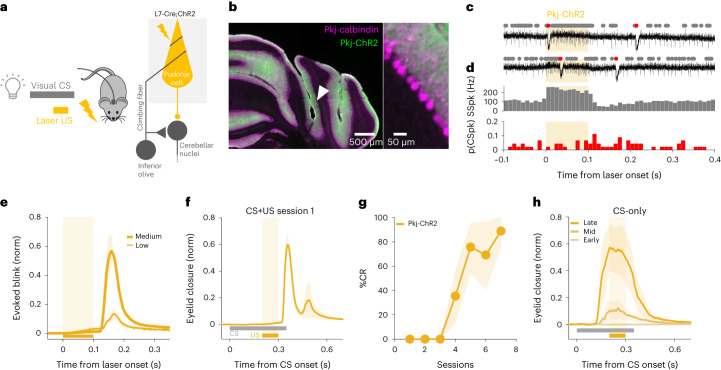
Optogenetic stimulation of Purkinje cells can substitute for a US to drive learning. **a**, Experimental scheme. L7-Cre;ChR2 mice were used to photostimulate Purkinje cells, which served as a US for conditioning. **b**, Example coronal section of cerebellar cortex indicating fiber placement in the eyelid area of the cerebellar cortex (white arrow) and labeling Purkinje cell ChR2 expression (green) and calbindin (magenta). Similar expression and fiber placement were observed in 11 mice. **c**, Example electrophysiological traces of Purkinje cell SSpks (gray dots) and CSpks (red dots) in response to Pkj-ChR2 laser stimulation (orange shading). **d**, Population histogram of SSpk rate (gray) and CSpk probability (p(CSpk)) (red; *n* = 44 trials, *N* = 2 cells from 2 mice) (see Extended Data Fig. 2d for statistics). **e**, Average eyelid closures ± s.e.m. (shadows) evoked by low and medium-power Pkj-ChR2 stimulation. Note the blink at stimulus offset. Peak amplitude of evoked blink: low versus medium power, ^*^P = 0.047, paired Student’s *t*-test (*N* = 4 mice). **f**, Average eyelid closures ± s.e.m. (shadows) on CS + US trials in the first training session showing the blink evoked by Pkj-ChR2-US laser stimulation (*N* = 4 mice). **g**, The %CR across training sessions ± s.e.m. (shadows) to a Pkj-ChR2 US (*N* = 4 mice, plotted as in Fig. 1j). **h**, Average eyelid traces ± s.e.m. (shadows) from CS-only trials of sessions 2, 4 and 7 of the experiments in **g**.

**Extended Data Fig. 2:**
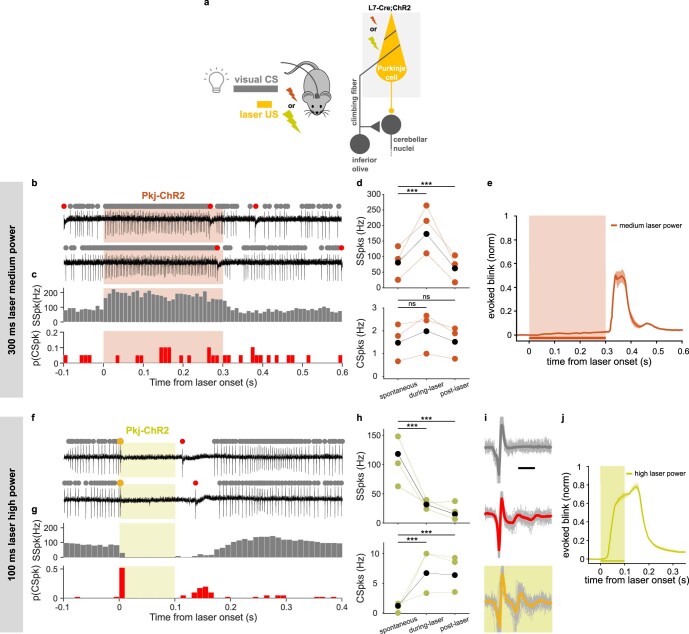
Varying intensity and duration of Purkinje cell optogenetic stimulation to dissociate stimulation onset, simple spike modulation, and evoked blinks. **a**, Experimental scheme for pairing a visual CS with optogenetic Purkinje cell-US. **b,c**, Example electrophysiological traces (b) and histogram (c) from a Purkinje cell with identified SSpk (grey dots) and CSpk (red) in response to 300 ms medium intensity Pkj-ChR2 laser stimulation in the cerebellar cortex (Pkj-ChR2-Ctx-med; corresponds to Fig. 3e-h). **d**, SSpk rate (top) and CSpk rate (bottom) pre-, during- and post- laser epochs for the recordings in (b,c) and Fig. 2d (N = 3 units from 2 mice). SSpks: spont. vs during laser, P = 0. 4.8e-7***, paired t-test; spont. vs after laser, P = 0.0003***, paired t-test. CSpks: spont. vs during laser, P = 0.22n.s., paired t-test; spont. vs after laser, P = 0.87n.s., paired t-test. Each orange circle and line represents a unit, linked through conditions; the black solid circles and line represent the average. **e**, Average eyelid closures ±SEM (shadows) to 300 ms Pkj-ChR2-Ctx medium intensity laser stimulation (N = 2 mice, shading represents laser stimulation). **f-j** Higher intensity Pkj-ChR2-Ctx laser stimulation was used to evoke a pause in simple spikes and a short-latency evoked blink at stimulus *onset* (corresponds to Fig. 3i-l). This stimulation elicited electrophysiological signatures of complex spike-like events at laser onset^48^ and, with a longer and more variable delay, at laser offset (likely due to rebound from release of Purkinje cell inhibition via olivo-cerebellar loop. **f,g**, SSpk and CSpk traces and histograms (N = 2 units from 2 mice). **h**, Same as in *d*, but for high intensity Pkj-ChR2-Ctx laser stimulation. SSpks: spont. vs during laser, P = 9.9e-17***, paired t-test; spont. vs after laser, P = 9.86e-20***, paired t-test. CSpks: spont. vs during laser, P = 1.335e-15***, paired t-test; spont. vs after laser, P = 2.45e-14***, paired t-test. **i**, SSpk (grey) and spontaneous CSpk (red) waveforms. Yellow trace represents complex spike-like events at laser stimulation onset; note the correspondence to spontaneous CSpk waveforms (red). **j**, Pkj-ChR2 laser stimulation at higher intensities yields a blink at stimulus *onset* (N = 3 mice, shading represents laser stimulation, ±SEM in shadows).

Optogenetic stimulation of Purkinje cells has previously been shown to instruct motor adaptation of limb and eye movements^28,47,48^. To investigate whether this was also true for delayed eyeblink conditioning, we placed an optical fiber at the eyelid region of the cerebellar cortex of transgenic mice expressing ChR2 under the L7 Purkinje cell-specific promoter (*L7-Cre;Chr2* mice; Fig. 2a,b). Consistent with previous studies^47,53,63–68^, in vivo electrophysiological recordings confirmed an increase in Purkinje cell SSpk activity at the onset of low–medium intensity optogenetic stimulation, followed by a slow decrease below the baseline firing rate on cessation of the stimulation, without significant changes in CSpk activity (Fig. 2c,d and Extended Data Fig. 2a–d).

The post-stimulation inhibition of Purkinje cell SSpks is consistently observed on optogenetic stimulation in vivo^47,63–68^, but not in vitro with synaptic activity blocked^69,70^, and probably reflects synaptically mediated network effects^71–73^. In the eyelid region of the cerebellar cortex, eyelid closures are associated with decreases in the Purkinje cell SSpk firing rate^55–58^. Consistent with this, and with previous optogenetic studies^53,56^, we found that optogenetic Purkinje cell stimulation with low–medium laser intensities resulted in eyelid closures on stimulus offset and that their amplitudes scaled as a function of laser intensity (Fig. 2e).

When a visual CS was consistently paired with a US consisting of optogenetic stimulation of Purkinje cells that drove increased SSpk activity at onset and a blink at laser offset (Pkj-ChR2; Fig. 2f), robust CRs gradually emerged (Fig. 2g,h). Rates of learning and CR amplitudes were comparable to those obtained with an airpuff US^53^ (%CR at last learning session: Pkj-ChR2 versus airpuff US controls in Extended Data Fig. 1o, *P* = 0.92 NS, Student’s *t*-test) and also with the CF-ChR2-US used in Fig. 1j (%CR at last learning session: Pkj-ChR2 versus CF-ChR2-IO, *P* = 0.24 NS, Student’s *t*-test; Pkj-ChR2 versus CF-ChR2-LE-IO, *P* = 0.75 NS, Student’s *t*-test). Notably, CRs were timed so that the peak eyelid closure coincided with the expected time of the onset of optogenetic stimulation (Fig. 2h).

The results of Fig. 2 suggest that direct optogenetic perturbation of Purkinje cells can substitute for an airpuff US to act as an instructive signal to drive eyeblink conditioning. However, they do not allow us to disentangle possible contributions of increases and/or decreases in Purkinje cell SSpks or evoked eyelid closures. In the next set of experiments, we systematically altered the temporal relationships between these candidate instructive signals by varying laser timing, duration and intensity.

### Onset of Purkinje cell stimulation drives learning

**Fig. 3:**
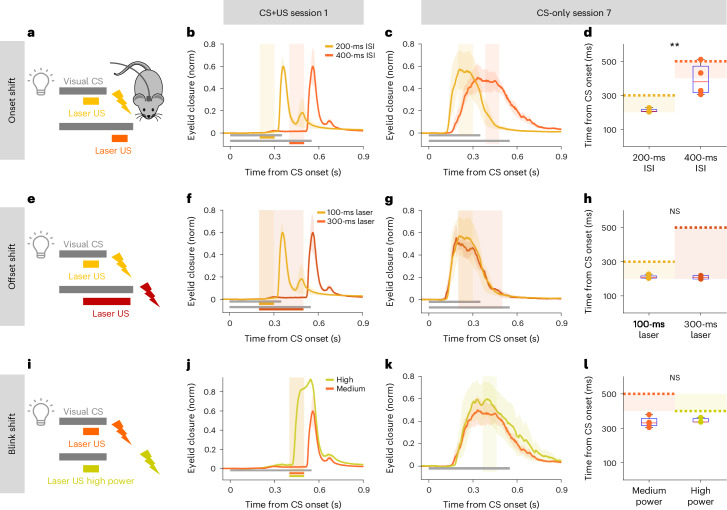
Learning evoked by optogenetic Purkinje cell stimulation is temporally coupled to stimulation onset and not evoked blinks or SSpk modulation. **a**,**e**,**i**, Schemes for Pkj-ChR2-US experiments in which stimulation onset timing, duration and intensity were varied systematically to dissociate candidate instructive signals (Extended Data Fig. 2). **a**, US onset shifts to obtain CS + US ISIs of 200 ms (yellow) or 400 ms (orange). **b**, CS + US trials before training, showing evoked blinks occurring at US offset in the two conditions (*N* = 4 mice for each ISI, ±s.e.m. in shadows). **c**, CS-only trials after training, showing the dependence of timing of learned eyelid closures on timing of US onset. **d**, Timing of peak eyelid closures occurring later for the longer ISI. Peak time: 200-ms versus 400-ms ISI, ^**^P = 0.009, two-sample Student’s *t*-test (4 versus 4 mice). Shaded rectangles indicate laser US duration and dashed lines blink onset. Each dot is one mouse; the box plots indicate median (center bar) and 25th to 75th percentiles (bottom and top borders), with whiskers extending to data extremes. **e**, US duration adjusted so that CS + US onset times were identical, but US offset (and blink) timing varied with respect to the CS. **f**, US-evoked blinks on CS + US trials occurring at stimulus offset (note temporal correspondence with blinks in **b**, ±s.e.m.) (Extended Data Fig. 2b–e). **g**,**h**, Learned CRs (**g**), and timing (**h**), showing timing dependence not on stimulus offset or the evoked blink, but, rather, stimulation onset (peak time: 100-ms versus 300-ms duration, *P* = 0.87 NS, two-sample Student’s *t*-test, 4 versus 2 mice). **i**,**j**, Laser intensity adjusted (**i**) to evoke a blink (**j**) (associated with a decrease in SSpks; Extended Data Fig. 2f–h) either at laser offset (orange ±s.e.m., as above) or, with higher intensities, at laser onset (lime green ±s.e.m., *N* = 3 mice). Laser US timings and durations were identical in the two conditions. **k**,**l**, Learned CRs (**k**) showing timing dependent only on time of stimulation onset and not varying with the timing of the evoked blink (**l**) (peak time: high versus medium laser power, *P* = 0.67 NS, two-sample Student’s *t*-test, 4 versus 3 mice) or the direction of SSpk modulation (Extended Data Fig. 2f–h).

The well-timed CRs observed in eyeblink conditioning are thought to be a consequence of plasticity mechanisms acting within the cerebellar cortex that associate postsynaptic calcium events (usually CSpks) in Purkinje cells with a particular set of parallel fiber inputs active within a particular temporal window from the onset of the CS^4,6,7,13,61,74^. We first asked whether learning to an optogenetic Pkj-ChR2 US would yield well-timed CRs to different CS–US intervals (Fig. 3a,b). Indeed, extending the interstimulus interval (ISI) between CS and Pkj-ChR2-US onset from 200 ms to 400 ms revealed appropriate corresponding shifts in CR timing (Fig. 3c,d).

Having thus established that learned responses to an optogenetic Purkinje cell (Pkj) US can be appropriately timed, we next varied the duration of laser stimulation (Fig. 3e and Extended Data Fig. 2b–e) to determine whether CRs were timed to match the increase in SSpk activity at the onset of Pkj-ChR2 stimulation, or its offset and the associated blink. To do this, we compared conditions in which the onset of Purkinje cell stimulation was presented at the same ISI relative to the CS, but the offset (and its respective blink) differed by 200 ms (Fig. 3e,f and Extended Data Fig. 2b–e). The CR amplitudes and timings were identical in the two groups (Fig. 3g,h). This result suggests that events associated with the onset of optogenetic stimulation, and not the decrease in the SSpk rate or the corresponding blink evoked at laser offset, are crucial for learning driven by optogenetic stimulation of Purkinje cells.

We next exploited the interrelationship of laser power, SSpk modulation and timing of the evoked blink to further disambiguate which consequences of the onset of optogenetic stimulation were responsible for optogenetically driven learning. At higher powers, laser stimulation induces a pause in Purkinje cell SSpk activity at laser onset (Extended Data Fig. 2f–h), probably owing to depolarization block^75,76^. Consistent with this and the well-established relationship between Purkinje cell SSpk inhibition and eyelid closures, we found that increasing laser intensity also led to a temporal shift in the timing of the optogenetically evoked blink—from laser offset to laser onset (Extended Data Fig. 2j). We took advantage of this feature to compare learning under conditions in which the timing and duration of the optogenetic US stimulation were identical, but laser power was adjusted to invert the direction of SSpk modulation and shift the timing of the evoked blink from laser offset to laser onset (Fig. 3i,j). As in the experiment presented in Fig. 3e–h, here, too, we found that CR timing depended only on the timing of laser onset, and not the timing of the evoked blink on the paired trials (Fig. 3k,l). This again suggests that the relevant instructive signal for learning occurs at the onset, and not the offset, of Purkinje cell optogenetic stimulation. Moreover, because the switch from increases in SSpk activity to pauses in SSpk activity is evoked by laser onset at high intensities (Extended Data Fig. 2f–h), it further dissociates laser onset from the modulation of Purkinje cell SSpks as the relevant instructive stimulus for learning.

Taken together, the results of Figs. 2 and 3 suggest that, although Purkinje cell optogenetic stimulation can substitute for an airpuff US to drive eyeblink conditioning, the effective instructive stimulus driving this learning is tightly linked to the onset of laser stimulation, but independent of either the direction of SSpk modulation or the blink that it evokes. One possible explanation for this finding would be if Pkj-ChR2 stimulation elevates dendritic calcium, triggering CSpk-like events that are capable of driving learning, as has been recently demonstrated for VOR adaptation^48^. Consistent with this possibility, we observed electrophysiological signatures of CSpk-like events at the onset of Pkj-ChR2 stimulation at higher stimulation intensities (Extended Data Fig. 2i).

**Fig. 4:**
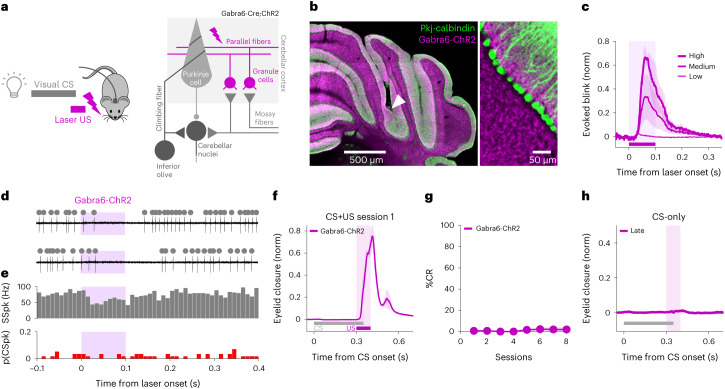
Optogenetic stimulation of cerebellar granule cells drives a blink but not learning. **a**, Experimental scheme. Gabra6-Cre;ChR2 mice were used to photostimulate cerebellar granule cells, which served as a US for conditioning. **b**, Example coronal sections (representative of six mice) of cerebellar cortex showing expression of ChR2 in granule cells (Gabra6-ChR2, magenta; Pkj-calbindin, green) and fiber placement in the eyeblink area of the cerebellar cortex (white arrow). **c**, Gabra6-ChR2 laser stimulation evoking intensity-dependent eyelid closures at stimulation onset (±s.e.m. in shadows). Peak amplitude of evoked blink: medium versus high power, ^*^P = 0.03, paired Student’s *t*-test (*N* = 6 mice). **d**, Example electrophysiological traces of Purkinje cell SSpk (gray dots) and CSpk (red dots) modulation to Gabra6-ChR2 laser stimulation (purple shading). **e**, Population histograms (*n* = 56 trials, *N* = 3 cells from 2 mice) showing decrease in SSpks (spontaneous versus laser, ^***^P = 1.28 × 10^−5^, paired Student’s *t*-test) and no change in CSpks (spontaneous versus laser: *P* = 1 NS, paired Student’s *t*-test) on laser stimulation. **f**, Average eyelid closures ± s.e.m. (shadows) on CS + US trials of the first training session showing the blink evoked by Gabra6-ChR2 laser stimulation (purple, *N* = 6 mice). **g**, The %CR across sessions ± s.e.m. in shadow (*N* = 6 mice). **h**, Average eyelid traces ± s.e.m. (shadows) from CS-only trials of the last training session showing no learning (purple, *N* = 6 mice).

If Pkj-ChR2-US stimulation drives eyeblink learning through the generation of dendritic CSpk-like events, then we would predict that Purkinje cell SSpk modulation driven by synaptic inputs rather than direct optogenetic stimulation might not be sufficient to induce learning, even if it were strong enough to evoke a blink. To test this prediction, we replaced direct Pkj-ChR2 stimulation with optogenetic stimulation of cerebellar granule cells, the axons of which form parallel fiber inputs to Purkinje cells (Gabra6-ChR2; Fig. 4a,b). As we have previously shown^53^, granule cell stimulation drives a blink at laser onset, consistent with net inhibition of Purkinje cells via molecular layer interneurons^77^ (Fig. 4c). Although this stimulation effectively modulated Purkinje cell SSpks (Fig. 4d,e) and drove a blink (Fig. 4c,f), it did not generate a CSpk-like-event (Fig. 4d,e), and pairing it with a visual CS did not result in learning (Fig. 4f–h).

The most parsimonious interpretation of the data presented in Figs. 2–4 and Extended Data Fig. 2 is that optogenetic stimulation of Purkinje cells drives eyeblink conditioning through cell-autonomous, CSpk-like events associated with stimulation onset^48^.

### Optogenetic inhibition of the IO blocks eyeblink conditioning

**Fig. 5:**
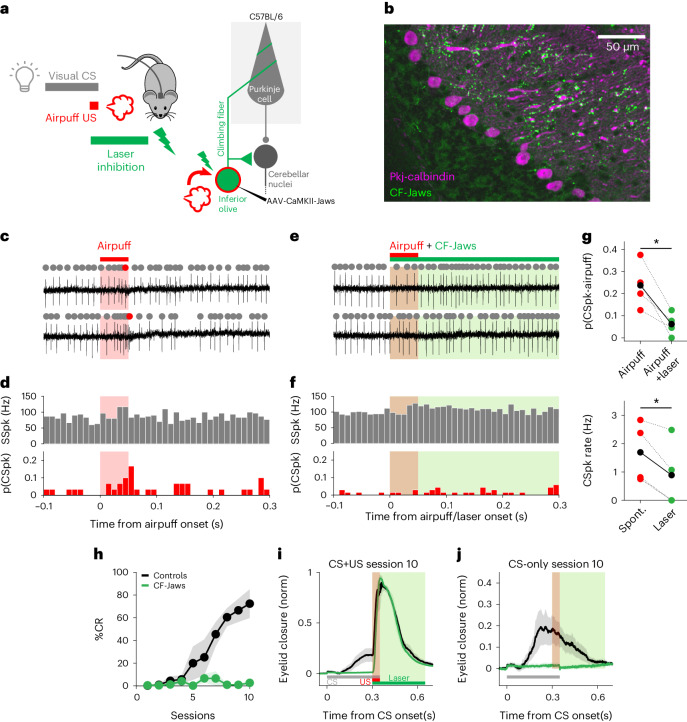
Inhibition of the IO that blocks airpuff US-driven CSpks eliminates eyeblink conditioning. **a**, Experimental scheme. Photoinhibition of CFs during airpuff US in CS + US trials. (Duration was randomized to avoid consistently timed rebound excitation; Methods.) Wild-type animals were injected with AAV-CamKII-Jaws in the IO where an optical fiber was also placed to photoinhibit CFs. **b**, Example sagittal section of cerebellar cortex. Similar expression was seen in eight mice. Jaws (green) is expressed in CF inputs to Purkinje cells (magenta). **c**, Two example electrophysiological traces from a Purkinje cell with identified SSpks (gray) and CSpks (red) in response to airpuff stimulation (red shading). **d**, Population histogram of SSpks (gray) and CSpks (red) (*n* = 29 trials, *N* = 4 units from 2 mice). **e**,**f**, Same as **c** and **d**, respectively, but paired with CF-Jaws laser inhibition (green; *n* = 68 trials, same units as in **c** and **d**). Spontaneous SSpk rate pre- and during laser epochs (*P* = 0.42 NS, paired Student’s *t*-test, *N* = 4 units). **g**, Top: average probability of CSpks in airpuff-only versus airpuff + laser trials (^*^P = 0.028, paired Student’s *t*-test, *N* = 4 units). Bottom: spontaneous CSpk rate pre- and during laser epochs (^*^P = 0.026, Student’s *t*-test, *N* = 4 units). Each circle represents a unit, linked through conditions by a dotted line; the black solid circles and line represent the average. **h**, The %CR across sessions ± s.e.m. (shadows) with and without CF-Jaws laser inhibition (green and black, respectively, *N* = 4 mice for both). The %CR at the last learning session: CF-Jaws versus controls, ^**^P = 0.0015, two-sample Student’s *t*-test. Control animals expressed Jaws in CFs but no laser was presented. **i**, Average eyelid closure traces ± s.e.m. (shadows) from CS + US trials of the last training session of the experiment shown in **h** revealing an absence of learning in the laser inhibition condition despite the intact unconditioned reflex to the airpuff US. The shaded rectangle indicates where in the trial the US (red) and the laser (green) appeared. **j**, Same as for **i**, but for CS-only trials. The shaded rectangles indicate where US and laser would have appeared.

We next used several complementary approaches to ask whether CF activity is required for delayed eyeblink conditioning to a sensory airpuff US. First, to inhibit CFs specifically at the time of the US, we injected a virus that allows for expression of the optogenetic inhibitor Jaws^78^ under control of the CaMKIIα promoter (AAV-CaMKII-Jaws) into the IO of wild-type mice (Fig. 5a). We observed selective labeling of neurons in the IO and CFs in the cerebellar cortex (Fig. 5b). As before, an optical fiber was placed in the dorsal accessory IO.

Photoinhibition of CFs blocked airpuff-driven CSpks (Fig. 5c–g) and also reduced spontaneous complex spiking during laser presentation (Fig. 5g). Moreover, laser inhibition of CFs at the time of the airpuff US completely prevented learning in CF-Jaws animals (Fig. 5h–j, green), whereas control mice expressing Jaws in CFs that did not receive laser inhibition learned normally (Fig. 5h–j, black). Notably, the learning impairment could not have been the result of an overall inability to respond to the US, because the reflexive blink to the airpuff US (UR) was intact (Fig. 5i). We conclude that optogenetic inhibition of CF signaling blocks learning to a natural, sensory airpuff US.

### Subtle reductions in CF signaling eliminate learning

The simultaneous global silencing of CFs through the optogenetic inhibition that we used in Fig. 5 is a dramatic manipulation that could have unexpected consequences for the olivocerebellar circuit. Our final experiment, however, provided additional, unexpected evidence that intact CF signaling is essential for associative cerebellar learning under natural conditions.

**Fig. 6:**
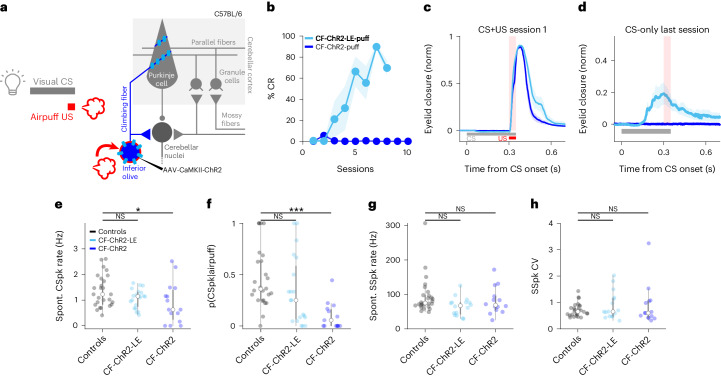
Moderate ChR2 expression is associated with subtle reductions in CF signaling and abolishes learning to a sensory US. **a**, Experimental scheme. A visual CS was paired with a sensory airpuff US in a traditional classic conditioning experiment in CF-ChR2 animals. **b**, CF-ChR2-puff animals, without any photostimulation, unable to learn to an airpuff US (blue, *N* = 6 mice), but recovered learning (CF-ChR2-LE-puff, light blue, *N* = 4 mice) on lowering ChR2 expression. Shadows correspond to ±s.e.m. (%CR at the last learning session: CF-ChR2-puff versus CF-ChR2-LE-puff, ^***^P = 7.75 × 10^−5^, two-sample Student’s *t*-test). **c**, Animals with both expression levels exhibiting robust UR blinks on CS + US trials (CF-ChR2-puff, blue, *N* = 6 mice and CF-ChR2-LE-puff, light blue, *N* = 4 mice, ±s.e.m. in shadows). **d**, Average eyelid traces ± s.e.m. (shadows) from CS-only trials of the last training session revealing no learning in CF-ChR2-puff animals (blue, *N* = 6 mice and CF-ChR2-LE-puff, light blue, *N* = 4 mice). **e**, Spontaneous (Spont.) CSpk firing rate for each Purkinje cell recorded from control (black, *N* = 26 cells from 4 mice), CF-ChR2-LE (light blue, *N* = 20 cells from 4 mice) and CF-ChR2 (blue, *N* = 15 units from 5 mice) mice. Controls versus CF-ChR2: ^*^P = 0.04, two-sample Student’s *t*-test (26 versus 15 cells); controls versus CF-ChR2-LE: *P* = 0.24 NS, two-sample Student’s *t*-test (26 versus 20 cells). **f**, Probability of an airpuff-evoked CSpk for each Purkinje cell recorded. Controls versus CF-ChR2: ^***^P = 0.00003, two-sample Student’s t-*t*est (26 versus 15 cells); controls versus CF-ChR2-LE: *P* = 0.16 NS, two-sample Student’s *t*-test (26 versus 20 cells). **g**,**h**, SSpk statistics for each Purkinje cell recorded from control (black), CF-ChR2-LE (light blue) and CF-ChR2 (blue) mice. **g**, SSpk spontaneous firing rate: controls versus CF-ChR2, *P* = 0.41 NS, two-sample Student’s *t*-test (26 versus 15 cells); controls versus CF-ChR2-LE, *P* = 0.07 NS, two-sample Student’s *t*-test (26 versus 20 cells). **h**, SSpk coefficient of variation (CV). Controls versus CF-ChR2: *P* = 0.33 NS, two-sample Student’s *t*-test (26 versus 15 cells); controls versus CF-ChR2-LE: *P* = 0.26 NS, two-sample Student’s *t*-test (26 versus 20 cells).

Surprisingly, we found that the CF-ChR2-expressing animals from Fig. 1, which learned well to an optogenetic US, were unable to learn in traditional eyeblink experiments using a sensory airpuff US, even in the absence of any laser stimulation (CF-ChR2-puff; Fig. 6a–d); the %CR at the last learning session: CF-ChR2-puff versus airpuff US controls in Extended Data Fig.1o, ^***^P = 1.98 × 10^−6^, Student’s *t*-test. In other words, simply expressing ChR2 in CFs completely blocked normal behavioral learning. This surprising result held true despite the facts that, as we have already shown: (1) ChR2 expression was specific to CFs in these mice (Fig. 1c and Extended Data Fig. 1); (2) spontaneous Purkinje cell CSpks were generally observed in these animals (Fig. 1d–f); (3) CSpks were readily evoked by CF optogenetic stimulation (Fig. 1d–f); (4) the mice displayed intact behavioral URs (blinks) to the airpuff (Fig. 6c), indicating intact sensory processing; and, of course, (5) CF-ChR2 animals had learned well to an optogenetic CF-ChR2-US (Fig. 1j,k).

Although we had used standard parameters for viral ChR2 expression^28,48,79^ and there was no obvious anatomical, physiological or behavioral indication of ChR2 overexpression, we next asked whether lower levels of ChR2 expression in CFs (CF-ChR2-LE; Fig. 1g–l) could restore learning to a sensory US. Remarkably, this fivefold reduction of viral titer fully restored the ability to learn to a sensory airpuff US (Fig. 6a–d); the %CR at the last learning session: CF-ChR2-LE-puff versus airpuff US controls in Extended Data Fig. 1o, *P* = 0.12 NS, Student’s *t*-test.

**Extended Data Fig. 3:**
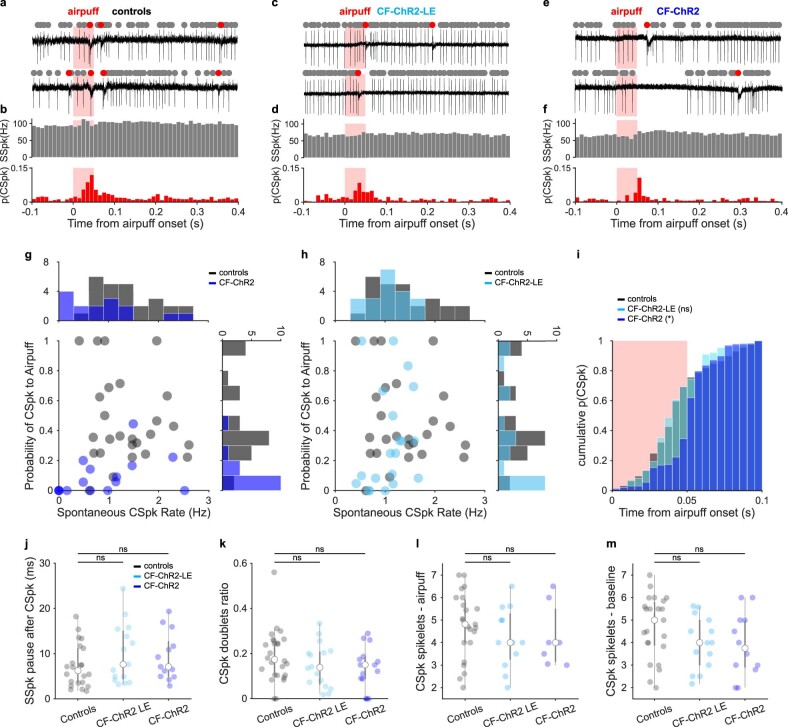
Purkinje cell responses to an airpuff stimulus in controls and CF-ChR2 animals with different expression levels. **a, b**, Example electrophysiological traces and population histograms (N = 26 units from 4 mice) of Purkinje cell SSpks (grey) and CSpks (red) from control mice in response to an airpuff. **c,d,** Same as *a,b* but for low ChR2-CF expression levels (CF-ChR2-LE; N = 20 units from 4 mice). **e,f**, Same as *c*,*d* but for standard CF-ChR2 expression (N = 15 units from 5 mice). **g**, p(CSpk) to airpuff vs. spontaneous CSpk rate for each Purkinje cell of controls vs. mice with standard CF-ChR2 expression (CF-ChR2). **h**, Same as *g*, but comparing controls vs. mice with low CF-ChR2 expression (CF-ChR2-LE). **i**, Normalized cumulative histogram of timing of the first CSpk after airpuff onset (grey: controls N = 26 cells from 4 animals; light blue, CF-ChR2-LE, N = 20 cells from 4 animals; blue: CF-ChR2 N = 15 cells from 5 mice); controls vs CF-ChR2, P = 0.03*, KS-test; controls vs CF-ChR2-LE, P =  0.67n.s., KS-test. Shaded rectangle indicates time of airpuff (red). **j**, Average pause in SSpks after a CSpk: controls vs CF-ChR2, P = 0.15n.s., two-sample t-test; controls vs CF-ChR2-LE, P = 0.24n.s., two- sample t-test. **k**, Average CSpk doublets (2 CSpks occurring within 200 ms of each other) ratio to total number of CSpks during spontaneous and airpuff epochs; controls vs CF-ChR2, P = 0.14n.s., two-sample t-test (15 cells in 5 mice); controls vs CF-ChR2-LE, P = 0.24n.s., two-sample t-test (20 cells in 4 mice). **l**,**m**, Average number of airpuff-driven and spontaneous CSpk spikelets, respectively. (*All two- sample t-test*) Airpuff: controls vs CF-ChR2, P = 0.5n.s., 15 cells in 5 mice; controls vs CF-ChR2- LE, P = 0.3n.s., 20 cells in 4 mice. Spont.: controls vs CF-ChR2, P = 0.1n.s. 15 cells in 5 mice; controls vs CF-ChR2-LE, P = 0.1n.s., 20 cells in 4 mice.

To understand how simply expressing ChR2 at moderate levels in CFs could have such a striking and selective impact on learning to a natural sensory US, we quantitatively compared electrophysiological recordings from Purkinje cells in CF-ChR2, CF-ChR2-LE and control mice (Fig. 6e–h and Extended Data Fig. 3). We analyzed spontaneous SSpks and CSpks as well as responses to airpuff stimuli delivered to the eye. In control conditions, CSpks are relatively infrequent, with a low average spontaneous firing rate and substantial variation across cells (Fig. 6e). We observed subtly lower spontaneous CSpk rates in Purkinje cells of CF-ChR2 mice compared with controls, whereas no significant reduction was observed in CF-ChR2-LE mice (Fig. 6e). Remarkably, we also found that some (4 of 15) units with moderate CF-ChR2 expression that showed clear, short-latency CSpks on CF-ChR2 stimulation did not exhibit any spontaneous CSpks throughout the duration of our recordings (Fig. 6e). This surprising finding indicates that the common method of identifying Purkinje cells based on the presence of spontaneous CSpks would obscure the consequences of CF-ChR2 expression for climbing fiber–Purkinje cell transmission.

We next analyzed the patterns of activity evoked by a sensory airpuff US (Extended Data Fig. 3a–f). There was a dramatic reduction in the probability of CSpks evoked by an airpuff stimulus in CF-ChR2 mice (Fig. 6f). This was true across the population of Purkinje cells that we recorded from these mice, including those with normal spontaneous CSpk rates (Extended Data Fig. 3g). In contrast, no systematic reduction in airpuff-evoked complex spiking was observed in the lower expression CF-ChR2-LE animals (Fig. 6f and Extended Data Fig. 3h). Furthermore, on trials in which an airpuff did evoke Purkinje cell CSpks, the responses were delayed in Purkinje cells recorded from CF-ChR2, but not CF-ChR2-LE, mice (Extended Data Fig. 3d,f,i).

Importantly, none of the differences in complex spiking observed in CF-ChR2 animals was associated with differences in Purkinje cell SSpks including average SSpk firing rate, coefficient of variation or the pause in SSpks after a CSpk (Fig. 6g,h and Extended Data Fig. 3j). We also found no differences in the proportion of CSpks occurring within 200 ms of each other (CSpk doublets^80^; Extended Data Fig. 3k) or in the number of spikelets within each CSpk waveform (Extended Data Fig. 3l,m).

## Discussion

**Fig. 7:**
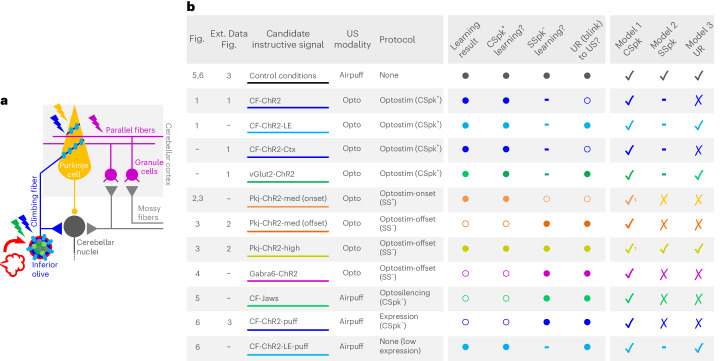
Summary of candidate instructive signals tested in the study and explanatory power of three models for cerebellar learning. **a**, Cerebellar circuit for eyeblink conditioning, highlighting the different strategies used in the present study. **b**, Summary table indicating the candidate instructive signal evaluated with each experiment, ordered and color coded by figure number. Ext. Data Fig., Extended Data Figure. For each candidate tested, the presence/absence of robust learning is indicated, followed by the predictions of CSpk^+^, SSpk^−^ or blink-driven models for learning. Closed circles represent ‘yes’ and open circles ‘no’. The last three columns assess the congruence between each model’s prediction and the learning result that was observed (check marks indicate congruence and Xs indicate lack of congruence; dashes indicate no prediction). Optostim, optogenetic stimulation.

The CF hypothesis for learning has dominated the cerebellar field for over 50 years^1–3^, yet definitive proof—or disproof—has remained elusive. Conflicting evidence, competing models and insufficiently precise tools for neural circuit dissection have sowed substantial controversy and confusion. In particular, although multiple experimental approaches have yielded data consistent with the theory, others have provided support for an alternative model, in which Purkinje cell SSpk modulation, rather than CSpks, provides critical instructive signals for learning^4,30,36,43^. Moreover, although sensorimotor errors that drive behavioral learning are often reflected in CF-driven Purkinje cell CSpk activity, the correlational nature of most of these studies, combined with the unusual spiking statistics of CSpks, has complicated a definitive interpretation of CSpks as instructive signals^34^. In the present study, we systematically manipulated distinct circuit elements to dissociate CF-driven CSpk signaling from Purkinje cell SSpk modulation and reflexive movements (Fig. 7). Our findings reveal excitatory CF inputs as necessary and sufficient instructive signals for associative cerebellar learning.

As has been recently shown for VOR adaptation^28,29,48^, we found that optogenetic stimulation of either CFs or Purkinje cells can substitute for a sensory US to drive eyeblink conditioning (Figs. 1 and 2 and Extended Data Fig. 1). In both cases, learning was independent of an evoked blink (Figs. 1–3 and Extended Data Figs. 1 and 2). However, additional experiments varying opto-Pkj-US laser intensity and duration revealed that learning to a Purkinje cell optogenetic US was temporally coupled to optogenetic US onset, regardless of the direction of Purkinje cell SSpk modulation or the timing of an evoked blink (Fig. 3). Further experiments in which Purkinje cell SSpk modulation was achieved indirectly, through optogenetic stimulation of granule cells, also failed to induce learning (Fig. 4). These findings suggest that optogenetic stimulation of Purkinje cells probably drives learning through the generation of CSpk-like dendritic calcium signals^48^ (Extended Data Fig. 2i), rather than through modulation of SSpk output. This could then also explain why CF-ChR2-expressing animals cannot access Purkinje cell instructive signals to achieve even minimal learning, as we have shown in Fig. 6.

Beyond demonstrating their sufficiency as instructive signals for learning, multiple aspects of our data point to the necessity of intact CF signaling for delayed eyeblink conditioning. First, Jaws-mediated optogenetic inhibition of CFs, specifically during the presentation of an airpuff US, completely abolished learning (Fig. 5). But perhaps the strongest evidence for the necessity of CF instructive signals came from our unexpected finding that simply expressing ChR2 in CFs—in the absence of any optical stimulation—reduced CSpk probability and completely obliterated learning to an airpuff US (Fig. 6). The complete absence of learning to a sensory US in these animals was particularly surprising, given the relative subtlety of the effects on complex spiking and the ability of these mice to learn to an optogenetic CF US (Fig. 1).

The exact mechanism of suppression of CF signaling by ChR2 expression remains to be determined. Our results point to a decrease in action potential generation within CFs themselves, rather than the postsynaptic generation of CSpks within Purkinje cells, because the probability of complex spiking was decreased, particularly in response to a sensory US, whereas CSpk waveforms were not affected (Extended Data Fig. 3l) and the ability to induce CSpks with optogenetic stimulation of CFs was spared. Such changes could also be associated with decreased synchrony across the CF population. Furthermore, decreases in CF activity may also alter the likelihood that CFs fire in bursts, or doublets^80^, under some conditions, although we did not see evidence for this in spontaneous CSpks (Extended Data Fig. 3k), and the drastic reduction in the evoked complex spiking that we observed made it impossible to address the potential further contribution of such a mechanism.

One of the most striking aspects of the ChR2 expression effect was its exquisite sensitivity to expression levels—a fivefold reduction in viral titer was enough to restore both normal complex spiking and learning to an airpuff US. It is well known that viral gene delivery^81^ and expression of ChR2 and other membrane proteins can alter neuronal morphology and physiology^82–85^ in ways that are still not fully understood. It is possible that IO neurons may be particularly vulnerable, for example, due to their high levels of electrical coupling^59,86,87^, which could explain the failure of many previous attempts to target CFs^25^. The use of adeno-associated virus (AAV)^81^ and/or of the CaMKIIa promoter may also have contributed, for instance by driving particularly strong expression levels^84^ or through perturbing endogenous CaMKII function in the IO^88^. However, the transgene itself appeared to be critical, because we did not observe a similar phenomenon when using the AAV with the CaMKIIa promoter to drive Jaws expression (Fig. 5h).

The discovery that small changes in ChR2 expression levels can have drastic behavioral consequences has important implications for experiments using optogenetic circuit dissection more broadly. For our purposes, CF-ChR2 expression provided an unexpectedly powerful and selective tool for reducing evoked CSpks, without affecting Purkinje cell simple spiking, while only subtly reducing spontaneous complex spiking. Still, the effects on complex spiking that we observed were not immediately obvious (Fig. 1) and depended on comprehensive quantitative analysis, which was possible only because cell-type-specific activity patterns in the cerebellar circuit and their relationship to relevant sensorimotor signals have previously been exceptionally well characterized. Thus, although we were able to exploit this unexpected effect as an unparalleled opportunity to assess the contributions of evoked CF signaling to cerebellar learning, our findings also highlight the major challenge of identifying circuit tools that allow neuroscientists to cleanly isolate and manipulate specific neural signals within complex networks.

Taken together, our results reconcile many previous, apparently contradictory, findings and suggest that CF-driven CSpk events provide essential instructive signals for cerebellar learning (Fig. 7).

Our findings also raise important questions about how sensorimotor errors are encoded in the cerebellum to support a full range of cerebellum-dependent behaviors. In particular, it is possible that parallel fiber inputs may provide instructive signals independent of CF input in some cases. For instance, whole-body movements like locomotion generate robust activation of mossy fiber inputs^89,90^. There is evidence that coincident input from spatially clustered parallel fibers can elicit dendritic calcium events in Purkinje cells^91–93^, which could drive cerebellar plasticity in the absence of CF inputs^94,95^. Although we were not able to induce such an effect via optogenetic stimulation of granule cells (Fig. 4), it remains possible that, during some forms of cerebellum-dependent learning, such as motor adaptation^28,96–98^, sufficiently high levels of parallel fiber activation could instruct parallel fiber plasticity.

Similarly, although our results reveal a necessary role for CF-driven Purkinje cell CSpks, they do not rule out a possible role for additional plasticity mechanisms in the cerebellar nuclei^99^, which may be important for some forms or components of learning, for instance across time scales^4,5,100–103^. Previous work has suggested that cerebellar learning may consist of multiple stages, with initial learning in the cerebellar cortex (driven mainly by CF inputs) leading to changes in Purkinje cell output that then sculpt plasticity in the cerebellar nuclei^4,5,100^. The relative contributions of cortical versus nuclear plasticity may vary across stages of learning or for different forms of cerebellar learning that progress on different time scales—from short-term motor adaptation over seconds and minutes^17,98,101^ to eyeblink conditioning, which takes days^102^, to long-term motor adaptation after prolonged wearing of prism goggles^103^, for example.

Regardless of possible contributions from additional mechanisms, our findings establish an absolute requirement for CF instructive signals in associative cerebellar learning and suggest that initial CSpk-driven plasticity could be an essential prerequisite for later stages of cerebellar learning to proceed.

## Methods

### Animals

All procedures were carried out in accordance with the European Union Directive 86/609/EEC and approved by the Champalimaud Centre for the Unknown Ethics Committee and the Portuguese Direção Geral de Veterinária (ref. nos. 0421/000/000/2015 and 0421/000/000/2020). Mice were kept in transparent cages with high-efficiency particulate air filters on a reversed 12-h light:12-h dark cycle, at 21 °C under relative humidity of 50% with free access to food and water. All procedures and experiments were performed in male and female mice aged approximately 12–14 weeks.

#### Mouse lines

Wild-type C57BL/6J mice were obtained from Jackson Laboratory (strain no. 000664). Selective ChR2 expression in Purkinje cells (*L7-Cre;ChR2*; Figs. 2 and 3 and Extended Data Fig. 2), granule cells (*Gabra6-Cre;ChR2*; Fig. 4) and glutamatergic neurons within the IO (*vGlut2-Cre;ChR2*; Extended Data Fig. 1) were obtained by crossing specific Cre driver lines with ChR2-EYFP-LoxP mice (strain no. 012569 from Jackson Laboratory^104^) to generate cell-type-specific, ChR2-expressing transgenic animals (Supplementary Table 1). Cre lines were: for Purkinje cells, L7-Cre strain no. 004146 from Jackson Laboratory^53,105^; for granule cells, Gabra6-Cre (MMRRC 000196-UCD^53,54,106,107^); and for glutamatergic neurons (within the IO), vGlut2-Cre (strain no. 016963 from Jackson Laboratory^60,108,109^).

### Surgical procedures

For all surgeries, animals were anesthetized with isoflurane (4% induction and 0.5–1.5% for maintenance), placed in a stereotaxic frame (David Kopf Instruments) and a custom-cut metal head plate was glued to the skull with dental cement (Super Bond, C&B). At the end of the surgery, mice were also administered a nonsteroidal anti-inflammatory and painkiller drug (carprofen). After all surgical procedures, mice were monitored and allowed ~1–2 d of recovery.

#### Viral injections

CFs were targeted^28,48^ by injecting 250 nl of AAV1.CaMKIIa.hChR2(H134R)-mCherry.WPRE.hGH (Addgene, catalog no. 26975 (ref. ^110^)) or AAV8.CamKII.Jaws-KGC.GFP.ER2-WPRE.SV40 (UPen, catalog no. AV-8-PV3637 (ref. ^78^)) into the left dorsal accessory IO, which has been previously implicated in eyeblink conditioning (rostrocaudal (RC) −6.3, mediolateral (ML) −0.5, dorsoventral (DV) 5.55 (refs. ^25,26,28^)). For the ChR2 virus, we initially diluted the stock virus 1:10 in artificial cerebrospinal fluid (aCSF) to yield a final titer of 1.31 × 10^12^ GC ml^−1^ (genome copies per ml) in line with previous studies^28^. For the low-expression (LE) conditions we diluted the virus an additional 5× to yield a final titer of 2,62 × 10^11^ GC ml^−1^. The Jaws virus was diluted in a ratio of 1:10 in aCSF to yield a final titer of 1.47 × 10^12^ GC ml^−1^. CF-ChR2 and CF-Jaws mice started the behavioral and electrophysiological experiments 6 weeks after injection to allow time for virus expression and stabilization^28^.

For optogenetic manipulations (Supplementary Table 1), optical fibers with 100-μm core diameter and 0.22 numerical aperture (NA; Doric lenses) were lowered into the brain through small craniotomies performed with a dental drill and positioned at either the right cerebellar cortical eyelid region (RC −5.7, ML +1.9, DV −1.5)^56,58,59^ or at the left dorsal accessory IO (RC −6.3, ML −0.5, DV −5.5), which has been previously implicated in eyeblink conditioning^25,26,111^. Correct fiber placement in both the cerebellar cortex and the IO was functionally verified before experiments by the presence of an evoked eyeblink in the right eye in response to moderate intensity laser stimulation (when possible; see below) and subsequently confirmed histologically. Only animals with good opsin expression and precise fiber targeting were kept in the study.

For in vivo electrophysiological recordings, a disposable 3-mm biopsy punch was used to perform a craniotomy over the right cerebellar cortical eyelid region (RC −5.7, ML +1.9 (refs. ^56,58,59^)). The craniotomy was covered with a 3-mm glass coverslip with four small holes where the electrode could pass through, and then by a silicon-based elastomer (Kwik-cast, WPI) that was easily removed just before recording sessions.

### Behavioral procedures

The experimental setup for eyeblink conditioning was based on previous work^53,54^. For all behavioral experiments, mice were head fixed and walking on a Fast-Trac Activity Wheel (Bio-Serv). A DC motor with an encoder (Maxon) was used to externally control the speed of the treadmill. Mice were habituated to the behavioral setup for at least 4 d before training, until they walked normally at the target speed of 0.1 m s^−1^ and displayed no external signs of distress. Eyelid movements of the right eye were recorded using a high-speed monochromatic camera (Genie HM640, Dalsa) to monitor a region of 172 × 160 pixel^2^ at 900 frames per s. We visually monitored whole-body movements via a webcam continuously throughout each experiment. Custom-written LabVIEW software, together with a NI PCIE-8235 frame grabber and a NI-DAQmx board (National Instruments), was used to synchronously trigger and control the hardware.

Acquisition sessions consisted of the presentation of 90 CS + US paired trials and 10 CS-only trials. The 100 trials were separated by a randomized intertrial interval of 10–15 s. Unless otherwise stated, CS and US onsets on CS + US paired trials were separated by a fixed ISI of 300 ms and both stimuli co-terminated. The CS was a white light LED positioned ~3 cm directly in front of the mouse. The sensory US was an airpuff (276 kpa, 50 ms) controlled by a Picospritzer (Parker) and delivered via a 27G needle positioned ~0.5 cm away from the cornea of the right eye of the mouse. Airpuff direction was adjusted for each session of each mouse so that the US elicited a strong reflexive eyeblink UR.

#### Behavioral analysis

Videos from each trial were analyzed offline with custom-written MATLAB (MathWorks) software^53^. The distance between eyelids was calculated frame by frame by thresholding the grayscale image and extracting the minor axis of the ellipse that delineated the eye. Eyelid traces were normalized for each session, from 0 (maximal opening of the eye throughout the session) to 1 (full eye closure achieved under airpuff treatment). Trials were classified as containing CRs if an eyelid closure with normalized amplitude >0.1 occurred >100 ms after CS onset and before US onset.

### Optogenetic stimulation and inhibition

Light from 473- or 594-nm lasers (LRS-0473or LRS-0594 DPSS, LaserGlow Technologies; excitation and inhibition, respectively) was controlled with custom-written LabView code. Predicted irradiance levels for the 100-μm diameter, 0.22-NA optical cannulae used in our study were calculated using the online platform: https://web.stanford.edu/group/dlab/optogenetics. All laser powers are comparable to those of previous studies^28,29,47,48,53,56,112^.

For Pkj-ChR2 (Figs. 2 and 3 and Extended Data Fig. 2), Gabra6-ChR2 (Fig. 4) and vGlut2-ChR2-IO (Extended Data Fig. 1) experiments, laser power was adjusted for each mouse and controlled for each experiment using a light power meter (Thorlabs) at the start and end of each session. For the Pkj-ChR2 experiments of Figs. 2 and 3a–h and Extended Data Fig. 2b–d, laser intensity was adjusted to elicit an intermediate eyelid closure, and no other body movements, at stimulus offset (1–3 mW, maximum irradiance of 95.5 mW mm^−2^). For the Pkj-ChR2-high (blink at laser onset) experiments of Fig. 3i–l and Extended Data Fig. 2e–h powers ranged from 8 mW to 12 mW, maximum irradiance of 381.8 mW mm^−2^. For the Gabra6-ChR2 experiments of Fig. 4, intensities were up to 6 mW, irradiance of 190.9 mW mm^−2^ (causing a blink at laser onset and no other body movements). For the vGlut2-ChR2-IO of Extended Data Fig. 1, laser power was adjusted to elicit a blink (and no other body movements) at laser onset (vGlut2-ChR2-IO: 1–3.3 mW, maximum predicted irradiance of 105 mW mm^−2^).

For the CF-ChR2 experiments of Fig. 1, because these animals did not blink (or present any other body movements) to laser stimulation (Fig. 1i), the power was set to 6 mW (maximum predicted irradiance of 190.9 mW mm^−2^). This power was confirmed with electrophysiology to reliably drive Purkinje cell CSpks. The same power was also used for all CF-ChR2-LE experiments, which exhibited a small eyelid twitch (and no other body movements) in response to laser stimulation (Fig. 1i).

When optogenetic stimulation was substituting for a sensory US, where possible we adjusted the timing (onset and duration) of the laser stimulation so that the reflexive blinks would most closely match those elicited by a sensory US (50-ms airpuff delivered to the eye). For Pkj-ChR2 and Gabra6-ChR2 experiments, 100-ms laser stimulation best elicited a blink similar to that of the airpuff. As Pkj-ChR2-med stimulation elicits a blink at the offset of laser stimulation, whereas GC-ChR2 elicits a blink at the onset of laser stimulation (owing to Purkinje cell inhibition via molecular layer interneurons), the onset of the laser stimulation was also adjusted specifically for those experiments. For the CF-ChR2 experiments of Fig. 1, as there was no laser-driven blink (Fig. 1i), we kept the 100-ms laser duration and matched the timing of laser stimulation/CSpk onset.

For the CF-Jaws experiments of Fig. 5, as these animals did not blink or present any other body movement to laser inhibition, the power was set to 6 mW (maximum predicted irradiance of 190.9 mW mm^−2^). This power was confirmed with electrophysiology to reliably block airpuff-driven Purkinje cell CSpks. For these inhibition experiments, laser started at the time of airpuff onset and laser duration were randomized between 300 ms and 400 ms to avoid consistently timed rebound excitation^78^.

### Electrophysiological recordings

All recordings were performed in vivo, in awake mice. Cell-attached, single-cell recordings were made using long-shanked borosilicate glass pipettes (Warner Instruments) pulled on a vertical puller (Narishige PC-100) and filled with saline solution (0.9% NaCl, typical resistances between 4 MΩ and 5 MΩ). An Optopatcher (A-M Systems) was used for simultaneous optogenetic stimulation and electrophysiological recordings. Laser light (with the same blue or yellow laser used for the behavioral optogenetic manipulations) was transmitted through an optic fiber (50-μm core diameter) inserted inside the glass pipette until it could fit, ~5 mm from the tip. The Optopatcher was oriented toward the cerebellar eyeblink region with a motorized four-axis micromanipulator (PatchStar, Scientifica). Craniotomies were filled with saline and connected to the ground reference using a silver-chloride pellet (Molecular Devices).

Recordings were performed with a Multiclamp 700B amplifier (Axon Instruments) in its voltage-clamp configuration, with a gain of 0.5 V nA^−1^ and low-pass Bessel filter with a 10-kHz cut-off. The current offset between the interior and exterior of the pipette was always kept neutral to avoid passive stimulation of the cells. All recordings were sampled at 25 kHz from the output signal of the amplifier using a NI-DAQmx board and Labview customized software. We observed subtly lower spontaneous CSpk rates (a typical criterion for identifying Purkinje cells in electrophysiological recordings), in Purkinje cells of CF-ChR2 mice compared with controls. As a result of this, for all CF-ChR2 (and CF-ChR2-LE) experiments, Purkinje cells were identified based on the presence of a laser-triggered CSpk rather than spontaneous CSpks, to avoid selection bias resulting from the absence of spontaneous complex spiking (Fig. 6). Spikes were sorted offline using customized Python code for SSpks and a modified Un’Eye neural network^113^ for CSpks.

### Histology

All experiments included histological verification of injection and fiber placement and transgene expression levels. After the experiments, animals were perfused transcardially with 4% paraformaldehyde and their brains removed. Brain sections (50 μm thick) were cut in a vibratome and stained for Purkinje cells with chicken anti-calbindin primary antibody (catalog no. 214006 SYSY) at dilution 1:300 and anti-chicken Alexa Fluor-488 (catalog no. 703-545-155) or Alexa Fluor-594 (catalog no. 703-585-155) secondary antibodies from Jackson Immunoresearch (both at dilution 1:800). General cell nuclear labeling was also made using DAPI. Brain sections were mounted on glass slides with mowiol mounting medium and imaged with ×5, ×10 or ×20 objectives. Brain slices from experiments where CFs were targeted were also imaged with an upright, confocal, laser point-scanning microscope (Zeiss LSM 710), using a ×10 or ×40 objective.

### Statistical analysis

Data are reported as mean ± s.e.m. and statistical analyses were performed using the Statistics toolbox in MATLAB. Two-sample, two-tailed or paired Student’s *t*-tests (specified in each case) were performed for all comparisons unless otherwise indicated. Differences were considered significant at: ^*^P < 0.05, ^**^P < 0.01 and ^***^P < 0.001. Data distribution was assumed to be normal but this was not formally tested. No statistical methods were used to predetermine sample sizes; sample sizes are similar to those reported in previous publications^53,54,56^. Data collection and analysis were not performed blind to the conditions of the experiments. Mice were randomly assigned to specific experimental groups without bias. No animals with validated histology or data points were excluded from analysis.

### Reporting summary

Further information on research design is available in the Nature Portfolio Reporting Summary linked to this article.

## Online content

Any methods, additional references, Nature Portfolio reporting summaries, source data, extended data, supplementary information, acknowledgements, peer review information; details of author contributions and competing interests; and statements of data and code availability are available at 10.1038/s41593-024-01594-7.

### Supplementary information

Supplementary Information Supplementary Tables 1 and 2.

Reporting Summary

## Data Availability

All datasets in the present paper are publicly available at https://gin.g-node.org/jerburi/ClimbFiber_InstructSignals (ref. ^114^).

## Code availability

All codes used for analysis are available from the lead author upon request.

## References

[1] Marr D (1969). A theory of cerebellar cortex. J. Physiol..

[2] Albus JS (1971). A theory of cerebellar function. Math. Biosci..

[3] Ito M (1972). Neural design of the cerebellar motor control system. Brain Res..

[4] Raymond JL, Lisberger SG, Mauk MD (1996). The cerebellum: a neuronal learning machine?. Science.

[5] Mauk MD, Donegan NH (1997). A model of Pavlovian eyelid conditioning based on the synaptic organization of the cerebellum. Learn. Mem..

[6] Medina JF, Garcia KS, Nores WL, Taylor NM, Mauk MD (2000). Timing mechanisms in the cerebellum: testing predictions of a large-scale computer simulation. J. Neurosci..

[7] De Zeeuw CI, Yeo CH (2005). Time and tide in cerebellar memory formation. Curr. Opin. Neurobiol..

[8] Raymond JL, Medina JF (2018). Computational principles of supervised learning in the cerebellum. Annu. Rev. Neurosci..

[9] Lisberger SG (2021). The rules of cerebellar learning: around the Ito hypothesis. Neuroscience.

[10] Ito M, Kano M (1982). Long-lasting depression of parallel fiber–Purkinje cell transmission induced by conjunctive stimulation of parallel fibers and climbing fibers in the cerebellar cortex. Neurosci. Lett..

[11] Schmolesky, M., Weber, J., de Zeeuw, C. & Hansel, C. The making of a complex spike: Ionic composition and plasticity. *Ann. NY Acad. Sci.*10.1111/j.1749-6632.2002.tb07581.x (2002). 10.1111/j.1749-6632.2002.tb07581.x 12582067

[12] Coesmans M, Weber JT, De Zeeuw CI, Hansel C (2004). Bidirectional parallel fiber plasticity in the cerebellum under climbing fiber control. Neuron.

[13] Carey MR (2011). Synaptic mechanisms of sensorimotor learning in the cerebellum. Curr. Opin. Neurobiol..

[14] Ramirez JE, Stell BM (2016). Calcium imaging reveals coordinated simple spike pauses in populations of cerebellar Purkinje cells. Cell Rep..

[15] Gilbert PF, Thach WT (1977). Purkinje cell activity during motor learning. Brain Res..

[16] Sears LL, Steinmetz JE (1990). Acquisition of classically conditioned-related activity in the hippocampus is affected by lesions of the cerebellar interpositus nucleus. Behav. Neurosci..

[17] Medina JF, Lisberger SG (2008). Links from complex spikes to local plasticity and motor learning in the cerebellum of awake-behaving monkeys. Nat. Neurosci..

[18] Rasmussen A, Jirenhed D-A, Wetmore DZ, Hesslow G (2014). Changes in complex spike activity during classical conditioning. Front. Neural Circuits.

[19] Kimpo RR, Rinaldi JM, Kim CK, Payne HL, Raymond JL (2014). Gating of neural error signals during motor learning. eLife.

[20] Ohmae S, Medina JF (2015). Climbing fibers encode a temporal-difference prediction error during cerebellar learning in mice. Nat. Neurosci..

[21] Stone LS, Lisberger SG (1990). Visual responses of Purkinje cells in the cerebellar flocculus during smooth-pursuit eye movements in monkeys. II. Complex spikes. J. Neurophysiol..

[22] Kahlon M, Lisberger SG (1996). Coordinate system for learning in the smooth pursuit eye movements of monkeys. J. Neurosci..

[23] Kim JJ, Krupa DJ, Thompson RF (1998). Inhibitory cerebello-olivary projections and blocking effect in classical conditioning. Science.

[24] Medina JF, Nores WL, Mauk MD (2002). Inhibition of climbing fibres is a signal for the extinction of conditioned eyelid responses. Nature.

[25] Kim OA, Ohmae S, Medina JF (2020). A cerebello-olivary signal for negative prediction error is sufficient to cause extinction of associative motor learning. Nat. Neurosci..

[26] Mauk MD, Steinmetz JE, Thompson RF (1986). Classical conditioning using stimulation of the inferior olive as the unconditioned stimulus. Proc. Natl Acad. Sci. USA.

[27] Steinmetz, J. E., Lavond, D. G. & Thompson, R. F. Classical conditioning in rabbits using pontine nucleus stimulation as a conditioned stimulus and inferior olive stimulation as an unconditioned stimulus. *Synapse* **3**, 225–233 (1989). 10.1002/syn.890030308 2718098

[28] Nguyen-Vu TDB (2013). Cerebellar Purkinje cell activity drives motor learning. Nat. Neurosci..

[29] Rowan MJM (2018). Graded control of climbing-fiber-mediated plasticity and learning by Inhibition in the cerebellum. Neuron.

[30] Ke MC, Guo CC, Raymond JL (2009). Elimination of climbing fiber instructive signals during motor learning. Nat. Neurosci..

[31] Zbarska S, Bloedel JR, Bracha V (2008). Cerebellar dysfunction explains the extinction-like abolition of conditioned eyeblinks after NBQX injections in the inferior olive. J. Neurosci..

[32] Schonewille M (2011). Reevaluating the role of LTD in cerebellar motor learning. Neuron.

[33] Popa LS, Streng ML, Hewitt AL, Ebner TJ (2016). The errors of our ways: understanding error representations in cerebellar-dependent motor learning. Cerebellum.

[34] Streng ML, Popa LS, Ebner TJ (2018). Complex spike wars: a new hope. Cerebellum.

[35] Sanger TD, Kawato M (2020). A cerebellar computational mechanism for delay conditioning at precise time intervals. Neural Comput..

[36] Miles FA, Lisberger SG (1981). Plasticity in the vestibulo-ocular reflex: a new hypothesis. Annu. Rev. Neurosci..

[37] Popa LS, Hewitt AL, Ebner TJ (2012). Predictive and feedback performance errors are signaled in the simple spike discharge of individual Purkinje cells. J. Neurosci..

[38] Lisberger SG, Fuchs AF (1978). Role of primate flocculus during rapid behavioral modification of vestibuloocular reflex. I. Purkinje cell activity during visually guided horizontal smooth-pursuit eye movements and passive head rotation. J. Neurophysiol..

[39] Kawato M, Furukawa K, Suzuki R (1987). A hierarchical neural-network model for control and learning of voluntary movement. Biol. Cybern..

[40] Du Lac S, Raymond JL, Sejnowski TJ, Lisberger SG (1995). Learning and memory in the vestibulo-ocular reflex. Annu. Rev. Neurosci..

[41] Raymond JL, Lisberger SG (1998). Neural learning rules for the vestibulo-ocular reflex. J. Neurosci..

[42] Carey MR, Medina JF, Lisberger SG (2005). Instructive signals for motor learning from visual cortical area MT. Nat. Neurosci..

[43] Shin S-L, Zhao GQ, Raymond JL (2014). Signals and learning rules guiding oculomotor plasticity. J. Neurosci..

[44] Albert ST, Shadmehr R (2016). The neural feedback response to error as a teaching signal for the motor learning system. J. Neurosci..

[45] Pugh JR, Raman IM (2009). Nothing can be coincidence: synaptic inhibition and plasticity in the cerebellar nuclei. Trends Neurosci..

[46] McElvain LE, Bagnall MW, Sakatos A, du Lac S (2010). Bidirectional plasticity gated by hyperpolarization controls the gain of postsynaptic firing responses at central vestibular nerve synapses. Neuron.

[47] Lee KH (2015). Circuit mechanisms underlying motor memory formation in the cerebellum. Neuron.

[48] Bonnan A, Rowan MMJ, Baker CA, Bolton MM, Christie JM (2021). Autonomous Purkinje cell activation instructs bidirectional motor learning through evoked dendritic calcium signaling. Nat. Commun..

[49] McCormick DA, Steinmetz JE, Thompson RF (1985). Lesions of the inferior olivary complex cause extinction of the classically conditioned eyeblink response. Brain Res..

[50] Luebke AE, Robinson DA (1994). Gain changes of the cat’s vestibulo-ocular reflex after flocculus deactivation. Exp. Brain Res..

[51] Zucca R, Rasmussen A, Bengtsson F (2016). Climbing fiber regulation of spontaneous Purkinje cell activity and cerebellum dependent blink responses. eNeuro.

[52] Lang EJ (2017). The roles of the olivocerebellar pathway in motor learning and motor control. a consensus paper. Cerebellum.

[53] Albergaria C, Silva NT, Pritchett DL, Carey MR (2018). Locomotor activity modulates associative learning in mouse cerebellum. Nat. Neurosci..

[54] Albergaria C, Silva NT, Darmohray DM, Carey MR (2020). Cannabinoids modulate associative cerebellar learning via alterations in behavioral state. eLife.

[55] Gauck V, Jaeger D (2000). The control of rate and timing of spikes in the deep cerebellar nuclei by inhibition. J. Neurosci..

[56] Heiney SA, Kim J, Augustine GJ, Medina JF (2014). Precise control of movement kinematics by optogenetic inhibition of Purkinje cell activity. J. Neurosci..

[57] Mostofi A, Holtzman T, Grout AS, Yeo CH, Edgley SA (2010). Electrophysiological localization of eyeblink-related microzones in rabbit cerebellar cortex. J. Neurosci..

[58] Steinmetz AB, Freeman JH (2014). Localization of the cerebellar cortical zone mediating acquisition of eyeblink conditioning in rats. Neurobiol. Learn. Mem..

[59] Van Der Giessen RS (2008). Role of olivary electrical coupling in cerebellar motor learning. Neuron.

[60] Hioki H (2003). Differential distribution of vesicular glutamate transporters in the rat cerebellar cortex. Neuroscience.

[61] Perrett SP, Ruiz BP, Mauk MD (1993). Cerebellar cortex lesions disrupt learning-dependent timing of conditioned eyelid responses. J. Neurosci..

[62] Chettih SN, McDougle SD, Ruffolo LI, Medina JF (2011). Adaptive timing of motor output in the mouse: the role of movement oscillations in eyelid conditioning. Front. Integr. Neurosci..

[63] Tsubota T, Ohashi Y, Tamura K, Sato A, Miyashita Y (2011). Optogenetic manipulation of cerebellar Purkinje cell activity in vivo. PLoS ONE.

[64] Canto CB, Witter L, De Zeeuw CI (2016). Whole-cell properties of cerebellar nuclei neurons in vivo. PLoS ONE.

[65] El-Shamayleh Y, Kojima Y, Soetedjo R, Horwitz GD (2017). Selective optogenetic control of Purkinje cells in monkey cerebellum. Neuron.

[66] Menardy F, Varani AP, Combes A, Léna C, Popa D (2019). Functional alteration of cerebello-cerebral coupling in an experimental mouse model of Parkinson’s disease. Cereb. Cortex.

[67] Jackman SL (2020). Cerebellar Purkinje cell activity modulates aggressive behavior. eLife.

[68] Bina L, Romano V, Hoogland TM, Bosman LWJ, De Zeeuw CI (2021). Purkinje cells translate subjective salience into readiness to act and choice performance. Cell Rep..

[69] Guo C (2016). Purkinje cells directly inhibit granule cells in specialized regions of the cerebellar cortex. Neuron.

[70] Guo C, Rudolph S, Neuwirth ME, Regehr WG (2021). Purkinje cell outputs selectively inhibit a subset of unipolar brush cells in the input layer of the cerebellar cortex. eLife.

[71] Orduz D, Llano I (2007). Recurrent axon collaterals underlie facilitating synapses between cerebellar Purkinje cells. Proc. Natl Acad. Sci. USA.

[72] Ankri L (2015). A novel inhibitory nucleo-cortical circuit controls cerebellar Golgi cell activity. eLife.

[73] Houck BD, Person AL (2015). Cerebellar premotor output neurons collateralize to innervate the cerebellar cortex. J. Comp. Neurol..

[74] Garcia KS, Steele PM, Mauk MD (1999). Cerebellar cortex lesions prevent acquisition of conditioned eyelid responses. J. Neurosci..

[75] Herman AM, Huang L, Murphey DK, Garcia I, Arenkiel BR (2014). Cell type-specific and time-dependent light exposure contribute to silencing in neurons expressing Channelrhodopsin-2. eLife.

[76] Chaumont J (2013). Clusters of cerebellar Purkinje cells control their afferent climbing fiber discharge. Proc. Natl Acad. Sci. USA.

[77] Grangeray-Vilmint A, Valera AM, Kumar A, Isope P (2018). Short-term plasticity combines with excitation–inhibition balance to expand cerebellar Purkinje cell dynamic range. J. Neurosci..

[78] Chuong AS (2014). Noninvasive optical inhibition with a red-shifted microbial rhodopsin. Nat. Neurosci..

[79] Aschauer DF, Kreuz S, Rumpel S (2013). Analysis of transduction efficiency, tropism and axonal transport of AAV serotypes 1, 2, 5, 6, 8 and 9 in the mouse brain. PLoS ONE.

[80] Titley HK, Kislin M, Simmons DH, Wang SS-H, Hansel C (2019). Complex spike clusters and false-positive rejection in a cerebellar supervised learning rule. J. Physiol..

[81] Suriano, C. M. et al. Adeno-associated virus (AAV) reduces cortical dendritic complexity in a TLR9-dependent manner. Preprint at *bioRxiv*10.1101/2021.09.28.462148 (2021).

[82] Zimmermann D (2008). Effects on capacitance by overexpression of membrane proteins. Biochem. Biophys. Res. Commun..

[83] Lin, J. Y. in *Optogenetics: Tools for Controlling and Monitoring Neuronal Activity* Progress in Brain Research Vol. 196 (eds Knöpfel, T. & Boyden, E. S.) 29–47 (Elsevier, 2012).

[84] Miyashita T, Shao Y, Chung J, Pourzia O, Feldman D (2013). Long-term Channelrhodopsin-2 (ChR2) expression can induce abnormal axonal morphology and targeting in cerebral cortex. Front. Neural Circuits.

[85] Liu M, Sharma AK, Shaevitz JW, Leifer AM (2018). Temporal processing and context dependency in *Caenorhabditis elegans* response to mechanosensation. eLife.

[86] Garden DLF (2018). Inferior olive HCN1 channels coordinate synaptic integration and complex spike timing. Cell Rep..

[87] Lefler Y, Yarom Y, Uusisaari MY (2014). Cerebellar inhibitory input to the inferior olive decreases electrical coupling and blocks subthreshold oscillations. Neuron.

[88] Bazzigaluppi P (2017). Modulation of murine olivary connexin 36 gap junctions by PKA and CaMKII. Front. Cell. Neurosci..

[89] Powell K, Mathy A, Duguid I, Häusser M (2015). Synaptic representation of locomotion in single cerebellar granule cells. eLife.

[90] Ishikawa T, Shimuta M, Häusser M (2015). Multimodal sensory integration in single cerebellar granule cells in vivo. eLife.

[91] Wang YT, Linden DJ (2000). Expression of cerebellar long-term depression requires postsynaptic clathrin-mediated endocytosis. Neuron.

[92] Roome CJ, Kuhn B (2018). Simultaneous dendritic voltage and calcium imaging and somatic recording from Purkinje neurons in awake mice. Nat. Commun..

[93] Roome CJ, Kuhn B (2020). Dendritic coincidence detection in Purkinje neurons of awake mice. eLife.

[94] Hartell NA (1996). Strong activation of parallel fibers produces localized calcium transients and a form of LTD that spreads to distant synapses. Neuron.

[95] Hartell NA (2002). Parallel fiber plasticity. Cerebellum.

[96] Herzfeld DJ, Kojima Y, Soetedjo R, Shadmehr R (2015). Encoding of action by the Purkinje cells of the cerebellum. Nature.

[97] Herzfeld DJ, Kojima Y, Soetedjo R, Shadmehr R (2018). Encoding of error and learning to correct that error by the Purkinje cells of the cerebellum. Nat. Neurosci..

[98] Darmohray DM, Jacobs JR, Marques HG, Carey MR (2019). Spatial and temporal locomotor learning in mouse cerebellum. Neuron.

[99] Broersen R (2023). Synaptic mechanisms for associative learning in the cerebellar nuclei. Nat. Commun..

[100] Kassardjian CD (2005). The site of a motor memory shifts with consolidation. J. Neurosci..

[101] Yang Y, Lisberger SG (2010). Learning on multiple timescales in smooth pursuit eye movements. J. Neurophysiol..

[102] Medina JF, Garcia KS, Mauk MD (2001). A mechanism for savings in the cerebellum. J. Neurosci..

[103] Lisberger SG (1994). Neural basis for motor learning in the vestibuloocular reflex of primates. III. Computational and behavioral analysis of the sites of learning. J. Neurophysiol..

[104] Madisen L (2012). A toolbox of Cre-dependent optogenetic transgenic mice for light-induced activation and silencing. Nat. Neurosci..

[105] Barski JJ, Dethleffsen K, Meyer M (2000). Cre recombinase expression in cerebellar Purkinje cells. Genesis.

[106] Fünfschilling U, Reichardt LF (2002). Cre-mediated recombination in rhombic lip derivatives. Genesis.

[107] Carey MR (2010). Presynaptic CB1 receptors regulate synaptic plasticity at cerebellar parallel fiber synapses. J. Neurophysiol..

[108] Fremeau RT (2001). The expression of vesicular glutamate transporters defines two classes of excitatory synapse. Neuron.

[109] Borgius L, Restrepo CE, Leao RN, Saleh N, Kiehn O (2010). A transgenic mouse line for molecular genetic analysis of excitatory glutamatergic neurons. Mol. Cell. Neurosci..

[110] Lee JH (2010). Global and local fMRI signals driven by neurons defined optogenetically by type and wiring. Nature.

[111] De Zeeuw CI, Wentzel P, Mugnaini E (1993). Fine structure of the dorsal cap of the inferior olive and its GAB aergic and non-Gabaergic input from the nucleus prepositus hypoglossi in rat and rabbit. J. Comp. Neurol..

[112] Arenkiel BR (2007). In vivo light-induced activation of neural circuitry in transgenic mice expressing Channelrhodopsin-2. Neuron.

[113] Markanday A (2020). Using deep neural networks to detect complex spikes of cerebellar Purkinje cells. J. Neurophysiol..

[114] Silva, N. T., Ramirez-Buriticá, J., Pritchett, D. L. & Carey, M. R. Data set for neural instructive signals for associative cerebellar learning. *G-node*10.12751/g-node.2wb3kg (2023).

