# Supplementary material for: Climbing fibers provide essential instructive signals for associative learning
Source: Nat Neurosci. 2024 Apr 2;27(5):940–51. doi: 10.1038/s41593-024-01594-7 (PMC11088996; doi:10.1038/s41593-024-01594-7)
Supplement: Supplementary file 1 — Supplementary Tables 1 and 2. [file 41593_2024_1594_MOESM1_ESM.pdf]

nature neuroscience

Article https://doi.org/10.1038/s41593-024-01594-7

Climbing fibers provide essential instructive signals for associative learning

In the format provided by the authors and unedited

Fig #	Experimental Condition	Expression strategy	Mouse genotype	Virus	Promoter	Cell type targeted	Fiber placement
1, 6, S1, S3	CF-ChR2	viral	wt(C57Bl6)	AAV-CamKII-ChR2 (titer ~10^{12})	CaMKII	CF	Ctx/IO
1, 6, S1, S3	CF-ChR2-LE	Viral – Low Expression	wt(C57Bl6)	AAV-CamKII-ChR2 (titer ~10^{11})	CaMKII	CF	IO
S1	vGlut2-ChR2-IO	transgenic	vGlut2-Cre;ChR2	-	vGlut2	IO-glut	IO
2, 3, S2	Pkj-ChR2	transgenic	L7-Cre;ChR2	-	L7	Pkj	Ctx
4	Gabra6-ChR2-Ctx	transgenic	Gabra6-Cre;ChR2	-	Gabra6	GC	Ctx
5	CF-Jaws	viral	wt(C57Bl6)	AAV-CamKII-Jaws (titer ~10^{12})	CaMKII	CF	IO

Supplementary Table 1. *Summary of genetic and anatomical targeting approaches used for selective targeting of individual cerebellar circuit elements.*

Fig #	Test	Independent Variable (IV)	IV levels	Dependent variable	p value	n (mice/cells)
1f	paired t-test	CF-ChR2 spont vs during laser	SSpk rate		0.22	4 cells, 2 mice
1f	paired t-test	CF-ChR2 spont vs during laser	CSpk rate		0.02	4 cells, 2 mice
1h	paired t-test	CF-ChR2-LE spont vs during laser	SSpk rate		0.107	8 cells, 2 mice
1h	paired t-test	CF-ChR2-LE spont vs during laser	CSpk rate		0.01	8 cells, 2 mice
1j	2 sample t-test	CF-ChR2 vs LE	% CR	last session of learning	0.115	7 vs 4 mice
1j	2 sample t-test	CF-ChR2 vs blue light ctls	% CR	last session of learning	1.7726e-04	7 vs 2 mice
1j	2 sample t-test	CF-ChR2-LE vs blue light ctls	% CR	last session of learning	4.0836e-05	4 vs 2 mice
1j, S1o	2 sample t-test	CF-ChR2 vs airpuff ctls	% CR	last session of learning	0.2939	7 vs 3 mice
1j, S1o	2 sample t-test	CF-ChR2-LE vs airpuff ctls	% CR	last session of learning	0.540	4 vs 3 mice
1m	paired t-test	CF-ChR2 300 vs 500ms	CR timing (peak amp)	last session of learning	0.014	4 mice
S1h	paired t-test	CF-ChR2-Ctx spont vs during laser	SSpk rate		0.002	15 cells, 5 mice
S1h	paired t-test	CF-ChR2-Ctx spont vs during laser	CSpk rate		1.81969e-06	15 cells, 5 mice
S1i	2 sample t-test	CF-ChR2 IO vs Ctx	% CR	last session of learning	0.71	3 vs 4 mice
S1k	2 sample t-test	CF-ChR2 IO vs Ctx	CR timing (peak amp)	last session of learning	0.37	3 vs 4 mice
S1o	2 sample t-test	vGlut2-ChR2 vs ChR2 ctls	% CR	last session of learning	0.94	3 vs 3 mice
2e	paired t-test	Pkj-ChR2 low vs medium	blink to laser (peak amp)		0.047	4 mice
2g, S1o	2 sample t-test	Pkj-ChR2 vs airpuff ctls	% CR	last session of learning	0.917	4 vs 3 mice
2g, 1j	2 sample t-test	Pkj-ChR2 vs CF-ChR2	% CR	last session of learning	0.2419	4 vs 7 mice
2g, 1j	2 sample t-test	Pkj-ChR2 vs CF-ChR2-LE	% CR	last session of learning	0.7545	4 vs 4 mice
3d	2 sample t-test	Pkj-ChR2 200 vs 400ms ISI	CR timing (peak amp)	last session of learning	0.009	4 vs 4 mice
3h	2 sample t-test	Pkj-ChR2 100 vs 300ms laser	CR timing (peak amp)	last session of learning	0.87	4 vs 2 mice
3l	2 sample t-test	Pkj-ChR2 high vs medium power	CR timing (peak amp)	last session of learning	0.67	4 vs 3 mice
S2d	paired t-test	Pkj-ChR2 spont vs during laser	SSpk rate		4.8e-7	3 cells, 2 mice
S2d	paired t-test	Pkj-ChR2 spont vs post laser	SSpk rate		0.0003	3 cells, 2 mice
S2d	paired t-test	Pkj-ChR2 spont vs during laser	CSpk rate		0.2228	3 cells, 2 mice
S2d	paired t-test	Pkj-ChR2 spont vs post laser	CSpk rate		0.8706	3 cells, 2 mice
S2h	paired t-test	Pkj-ChR2 spont vs during laser high	SSpk rate		9.911e-17	3 cells, 2 mice
S2h	paired t-test	Pkj-ChR2 spont vs post laser high	SSpk rate		9.86e-20	3 cells, 2 mice
S2h	paired t-test	Pkj-ChR2 spont vs during laser high	CSpk rate		1.335e-15	3 cells, 2 mice
S2h	paired t-test	Pkj-ChR2 spont vs post laser high	CSpk rate		2.44996e-14	3 cells, 2 mice
4c	paired t-test	GC-ChR2 medium vs high	blink to laser (peak amp)		0.027	4 mice
4e	paired t-test	Gabra6-ChR2 spont vs during laser	SSpk rate		1.28227e-05	3 cells, 2 mice
4e	paired t-test	Gabra6-ChR2 spont vs during laser	CSpk rate		1	3 cells, 2 mice
5g	paired t-test	CF-Jaws spont vs laser	SSpk rate		0.42	4 cells, 2 mice
5g	paired t-test	CF-Jaws spont vs laser	CSpk rate		0.026	4 cells, 2 mice
5g	paired t-test	CF-Jaws airpuff vs airpuff + laser	CSpk probab		0.028	4 cells, 2 mice
5h	2 sample t-test	CF-Jaws laser inhibition vs Jaws ctls	% CR	last session of learning	0.0015	4 vs 4 mice
6b	2 sample t-test	CF-ChR2-puff vs CF-ChR2-LE-puff	% CR	last session of learning	7.7482e-05	4 vs 4 mice
6b, S1o	2 sample t-test	CF-ChR2-LE-puff vs airpuff ctls	% CR	last session of learning	0.1215	4 vs 3 mice
6b, S1o	2 sample t-test	CF-ChR2-puff vs airpuff ctls	% CR	last session of learning	1.9849e-06	4 vs 3 mice
6e	2 sample t-test	CF-ChR2 vs controls	CSpk rate		0.04	26 vs 15 cells
6e	2 sample t-test	CF-ChR2-LE vs controls	CSpk rate		0.24	26 vs 20 cells
6f	2 sample t-test	CF-ChR2 vs controls	CSpk probab		0.00003	26 vs 15 cells
6f	2 sample t-test	CF-ChR2-LE vs controls	CSpk probab		0.16	26 vs 20 cells
6g	2 sample t-test	CF-ChR2 vs controls	SSpk rate		0.41	26 vs 15 cells
6g	2 sample t-test	CF-ChR2-LE vs controls	SSpk rate		0.07	26 vs 20 cells
6h	2 sample t-test	CF-ChR2 vs controls	SSpk CV		0.33	26 vs 15 cells
6h	2 sample t-test	CF-ChR2-LE vs controls	SSpk CV		0.26	26 vs 20 cells
S3i	2 sample ks-test	CF-ChR2 vs controls	CSpk timing		0.03	15 cells, 5 mice
S3i	2 sample ks-test	CF-ChR2-LE vs controls	CSpk timing		0.67	20 cells, 4 mice
S3j	2 sample t-test	CF-ChR2 vs controls	SSpk pause		0.15	15 cells, 5 mice
S3j	2 sample t-test	CF-ChR2-LE vs controls	SSpk pause		0.24	20 cells, 4 mice
S3k	2 sample t-test	CF-ChR2 vs controls	CSpk spikelets spont		0.1	15 cells, 5 mice
S3k	2 sample t-test	CF-ChR2-LE vs controls	CSpk spikelets spont		0.1	20 cells, 4 mice
S3l	2 sample t-test	CF-ChR2 vs controls	CSpk spikelets airpuff		0.5	15 cells, 5 mice
S3l	2 sample t-test	CF-ChR2-LE vs controls	CSpk spikelets airpuff		0.3	20 cells, 4 mice
S3m	2 sample t-test	CF-ChR2 vs controls	CSpk doublets		0.14	15 cells, 5 mice
S3m	2 sample t-test	CF-ChR2-LE vs controls	CSpk doublets		0.24	20 cells, 4 mice

Supplementary Table 2: *Information about statistical tests. All tests were two-sided.*
