# Supplementary material for: Climbing fibers provide essential instructive signals for associative learning
Source: Nat Neurosci. 2024 Apr 2;27(5):940–51. doi: 10.1038/s41593-024-01594-7 (PMC11088996; doi:10.1038/s41593-024-01594-7)
Supplement: Supplementary file 2 — Reporting Summary [file 41593_2024_1594_MOESM2_ESM.pdf]

nature portfolio

Corresponding author(s): Megan R. Carey, Dominique L. Pritchett.

Last updated by author(s): Jan 12, 2024

Reporting Summary

Nature Portfolio wishes to improve the reproducibility of the work that we publish. This form provides structure for consistency and transparency in reporting. For further information on Nature Portfolio policies, see our Editorial Policies and the Editorial Policy Checklist.

Statistics

For all statistical analyses, confirm that the following items are present in the figure legend, table legend, main text, or Methods section.

n/a	Confirmed	
☐	☒	The exact sample size (*n*) for each experimental group/condition, given as a discrete number and unit of measurement
☐	☒	A statement on whether measurements were taken from distinct samples or whether the same sample was measured repeatedly
☐	☒	The statistical test(s) used AND whether they are one- or two-sided<br>*Only common tests should be described solely by name; describe more complex techniques in the Methods section.*
☐	☒	A description of all covariates tested
☐	☒	A description of any assumptions or corrections, such as tests of normality and adjustment for multiple comparisons
☐	☒	A full description of the statistical parameters including central tendency (e.g. means) or other basic estimates (e.g. regression coefficient) AND variation (e.g. standard deviation) or associated estimates of uncertainty (e.g. confidence intervals)
☐	☒	For null hypothesis testing, the test statistic (e.g. *F*, *t*, *r*) with confidence intervals, effect sizes, degrees of freedom and *P* value noted<br>*Give P values as exact values whenever suitable.*
☒	☐	For Bayesian analysis, information on the choice of priors and Markov chain Monte Carlo settings
☒	☐	For hierarchical and complex designs, identification of the appropriate level for tests and full reporting of outcomes
☒	☐	Estimates of effect sizes (e.g. Cohen's *d*, Pearson's *r*), indicating how they were calculated

Our web collection on statistics for biologists contains articles on many of the points above.

Software and code

Policy information about availability of computer code

Data collection	All data was collected using custom-written code in LabView. Code is available from the corresponding author upon request.
Data analysis	All data was analyzed using custom-written code in Matlab (MathWorks) and Python version 3.6. All code is available from the corresponding author upon request.

For manuscripts utilizing custom algorithms or software that are central to the research but not yet described in published literature, software must be made available to editors and reviewers. We strongly encourage code deposition in a community repository (e.g. GitHub). See the Nature Portfolio guidelines for submitting code & software for further information.

Data

Policy information about availability of data

All manuscripts must include a data availability statement. This statement should provide the following information, where applicable:

- Accession codes, unique identifiers, or web links for publicly available datasets
- A description of any restrictions on data availability
- For clinical datasets or third party data, please ensure that the statement adheres to our policy

All datasets in this paper are publicly available at: https://gin.g-node.org/jerburi/ClimbFiber_InstructSignals (ref. 114).

Research involving human participants, their data, or biological material

Policy information about studies with human participants or human data. See also policy information about sex, gender (identity/presentation), and sexual orientation and race, ethnicity and racism.

Reporting on sex and gender	n/a
Reporting on race, ethnicity, or other socially relevant groupings	n/a
Population characteristics	n/a
Recruitment	n/a
Ethics oversight	n/a

Note that full information on the approval of the study protocol must also be provided in the manuscript.

Field-specific reporting

Please select the one below that is the best fit for your research. If you are not sure, read the appropriate sections before making your selection.

☒ Life sciences ☐ Behavioural & social sciences ☐ Ecological, evolutionary & environmental sciences

For a reference copy of the document with all sections, see nature.com/documents/nr-reporting-summary-flat.pdf

Life sciences study design

All studies must disclose on these points even when the disclosure is negative.

Sample size	No statistical methods were used to predetermine sample sizes; sample sizes are similar to those reported in previous publications, as in Catarina Albergaria N Tatiana Silva Dana M Darmohray Megan R Carey (2020) Cannabinoids modulate associative cerebellar learning via alterations in behavioral state. eLife 9:e61821.<br>Statistical tests were run after data collection.
Data exclusions	No data points were excluded.
Replication	Findings were replicated successfully across separate sets of animals in our experiments (details in methods and in the statistics summary found in Supplementary Table 2).
Randomization	Mice were assigned to specific experimental groups without bias.
Blinding	Due to genotype requirements no blinding was used but mice were assigned to specific experimental groups without bias and no animals were excluded.

Reporting for specific materials, systems and methods

We require information from authors about some types of materials, experimental systems and methods used in many studies. Here, indicate whether each material, system or method listed is relevant to your study. If you are not sure if a list item applies to your research, read the appropriate section before selecting a response.

Materials & experimental systems

n/a	Involved in the study	
☐	☒	Antibodies
☒	☐	Eukaryotic cell lines
☒	☐	Palaeontology and archaeology
☐	☒	Animals and other organisms
☒	☐	Clinical data
☒	☐	Dual use research of concern
☒	☐	Plants

Methods

n/a	Involved in the study	
☒	☐	ChIP-seq
☒	☐	Flow cytometry
☒	☐	MRI-based neuroimaging

Antibodies

Antibodies used	- Chicken anti-calbindin primary antibody #214006 from Synaptic Systems (dilution 1:500, ~0.1 ml per slice).<br>- Anti-chicken Alexa488 #703-545-155 secondary antibodies, from Jackson Immunoresearch (dilution 1:800, ~0.1 ml per slice).<br>- Anti-chicken Alexa594 #703-585-155 secondary antibodies, from Jackson Immunoresearch (dilution 1:800, ~0.1 ml per slice).

Validation	PRIMARY ANTIBODIES:<br>Chicken anti-calbindin #214006, Synaptic Systems<br>Amot and Yap1 regulate neuronal dendritic tree complexity and locomotor coordination in mice. Published and validated in IHC. Tested species: mouse. PubMed ID: 31042703

Animals and other research organisms

Policy information about studies involving animals; ARRIVE guidelines recommended for reporting animal research, and Sex and Gender in Research

Laboratory animals	Female and male mice obtained from The Jackson Laboratory (WT C57BL/6J #000664; ChR2-EYFP-LoxP #012569; L7-Cre #004146; vGlut2-Cre #016963) and MMRRC (Gabra6-Cre #000196-UCD), with approximately 12-14 weeks of age at time of surgery, were used.
Wild animals	The study did not involve wild animals.
Reporting on sex	Both females and males were used indiscriminately, since no sex effect was observed.
Field-collected samples	The study did not involve samples collected from the field.
Ethics oversight	All procedures were carried out in accordance with the European Union Directive 86/609/EEC and approved by the Champalimaud Centre for the unknown Ethics Committee and the Portuguese Direção Geral de Veterinária (Ref. No. 0421/000/000/2015 and 0421/000/000/2020).

Note that full information on the approval of the study protocol must also be provided in the manuscript.
